# Adherence to the 2018 World Cancer Research Fund (WCRF)/American Institute for Cancer Research (AICR) Cancer Prevention Recommendations and risk of 14 lifestyle-related cancers in the UK Biobank prospective cohort study

**DOI:** 10.1186/s12916-023-03107-y

**Published:** 2023-11-28

**Authors:** Fiona C. Malcomson, Solange Parra-Soto, Frederick K. Ho, Liya Lu, Carlos Celis-Morales, Linda Sharp, John C. Mathers

**Affiliations:** 1 Human Nutrition & Exercise Research Centre, Centre for Healthier Lives, Population Health Sciences Institute, Newcastle University, Newcastle Upon Tyne, UK; 2 Centre for Cancer, Population Health Sciences Institute, Newcastle University, Newcastle Upon Tyne, UK; 3 School of Health and Wellbeing, University of Glasgow, Glasgow, UK; 4 School of Cardiovascular and Medical Sciences, University of Glasgow, Glasgow, UK; 5 Department of Nutrition and Public Health, Universidad del Bío-Bío, Chillan, Chile; 6 Human Performance Lab, Education, Physical Activity and Health Research Unit, University Católica del Maule, Talca, Chile

**Keywords:** Cancer Prevention Recommendations, Lifestyle, Cancer incidence

## Abstract

**Background:**

The World Cancer Research Fund (WCRF)/American Institute for Cancer Research (AICR) Cancer Prevention Recommendations are lifestyle-based recommendations which aim to reduce cancer risk. This study investigated associations between adherence, assessed using a standardised scoring system, and the risk of all cancers combined and of 14 cancers for which there is strong evidence for links with aspects of lifestyle in the UK.

**Methods:**

We used data from 94,778 participants (53% female, mean age 56 years) from the UK Biobank. Total adherence scores (range 0–7 points) were derived from dietary, physical activity, and anthropometric data. Associations between total score and cancer risk (all cancers combined; and prostate, breast, colorectal, lung, uterine, liver, pancreatic, stomach, oesophageal, head and neck, ovarian, kidney, bladder, and gallbladder cancer) were investigated using Cox proportional hazard models, adjusting for age, sex, deprivation index, ethnicity, and smoking status.

**Results:**

Mean total score was 3.8 (SD 1.0) points. During a median follow-up of 8 years, 7296 individuals developed cancer. Total score was inversely associated with risk of all cancers combined (HR: 0.93; 95%CI: 0.90–0.95 per 1-point increment), as well as breast (HR: 0.90; 95%CI: 0.86–0.95), colorectal (HR: 0.90; 95%CI: 0.84–0.97), kidney (HR: 0.82; 95%CI: 0.72–0.94), oesophageal (HR: 0.84; 95%CI: 0.71–0.98), ovarian (HR: 0.76; 95%CI: 0.65–0.90), liver (HR: 0.78; 95%CI: 0.63–0.97), and gallbladder (HR: 0.70; 95%CI: 0.53–0.93) cancers.

**Conclusions:**

Greater adherence to lifestyle-based recommendations was associated with reduced risk of all cancers combined and of breast, colorectal, kidney, oesophageal, ovarian, liver, and gallbladder cancers. Our findings support compliance with the Cancer Prevention Recommendations for cancer prevention in the UK.

**Supplementary Information:**

The online version contains supplementary material available at 10.1186/s12916-023-03107-y.

## Background

Lifestyle factors, including diet, physical activity, and body composition, are associated with the risk of several common cancers including breast and colorectal cancer. In the UK, approximately 40% of cancer cases are attributable to modifiable risk factors such as tobacco smoking, overweight and obesity, insufficient dietary fibre intake, and alcohol consumption [1]. To reduce the risk of cancer (and other non-communicable diseases), the World Cancer Research Fund (WCRF)/American Institute for Cancer Research (AICR) published ten Cancer Prevention Recommendations, updated in 2018, that encourage a healthy lifestyle pattern, including eating a healthy diet, maintaining a healthy body weight, and undertaking adequate physical activity [2].

Several studies have investigated associations between adherence to the WCRF/AICR Cancer Prevention Recommendations and the risk of cancer diagnosis, cancer survival, and other health-related outcomes. However, most of the studies to date have assessed adherence to the earlier, 2007, version of the recommendations which differs in terms of the recommendations themselves (i.e. the recommendation to ‘eat less salt’ has been removed and a recommendation to ‘limit consumption of sugar-sweetened drinks’ has been added), as well as how adherence to the recommendations is assessed (e.g. a cut-off of consuming at least 25 g per day of dietary fibre was used to meet the 2007 sub-recommendation which has been increased to at least 30 g per day in the 2018 version). A systematic review and meta-analysis of the evidence for associations between adherence to the 2007 Cancer Prevention Recommendations and cancer prevention and other health outcomes concluded that greater adherence was associated with reduced risk of breast, colorectal, and lung cancer, as well as of cancer-specific and overall mortality [3]. The authors reported significant methodological heterogeneity across studies, due mainly to the different approaches used to assess adherence to the recommendations including the number of recommendations included and the cut-offs used to assess adherence to each recommendation [3]. To address this issue and to allow greater consistency and comparability between studies, in 2019, Shams-White and colleagues created a standardised scoring system (the ‘2018 WCRF/AICR Score’) which operationalises seven (and an optional eighth) of the ten recommendations, and encouraged researchers to apply the scoring system in studies across cohorts worldwide [4]. To date, a limited number of studies have operationalised this standardised scoring system to measure adherence to the 2018 Cancer Prevention Recommendations. Most have investigated overall cancer incidence or limited consideration to the more common cancers such as breast [5], colorectal [6], and prostate [7] cancers. In addition, studies have varied in how they have operationalised the 2018 WCRF/AICR Score, with few fully following the scoring system [8]. Further, these studies have largely been conducted in the USA [9, 10], Spain [5, 7], and Sweden [11] and generalisability of the findings to other countries, including the UK, is uncertain.

The aim of this study was to investigate—for the first time in the UK—associations between the score, derived by fully operationalising the 2018 WCRF/AICR Score, and incidence of multiple cancers. Using data from the UK Biobank prospective cohort study, we assessed the risk of all invasive cancers as well as of 14 specific cancers (prostate, breast, colorectal, lung, uterine, kidney, bladder, ovarian, pancreatic, head and neck, oesophageal, stomach, liver, and gallbladder) for which there is strong evidence for a relationship with aspects of diet, nutrition, and/or physical activity [2].

## Methods

### The UK Biobank Study

The UK Biobank is a prospective cohort study which recruited > 500,000 participants aged 37–73 years, 56% female, from 22 centres across England, Scotland, and Wales between 2006 and 2010. The study protocol can be found at https://www.ukbiobank.ac.uk/media/gnkeyh2q/study-rationale.pdf. During the baseline study visit at an assessment centre, a touchscreen questionnaire was used to collect self-reported data on sociodemographic factors, diet, and general health, and anthropometric measurements were made.

### Ethics

All UK Biobank participants provided informed consent. The study was conducted in accordance with the Declaration of Helsinki and was approved by the North West Multi-centre Research Ethics Committee (REC reference: 12/NW/03820).

### Score to measure adherence to the 2018 WCRF/AICR Cancer Prevention Recommendations

We assessed adherence to the 2018 WCRF/AICR Cancer Prevention Recommendations by fully operationalising the seven-component version of the 2018 WCRF/AICR Score [4]. The eighth, and optional, ‘Breastfeeding’ component of the score was not operationalised due to a lack of data. A detailed description of the operationalisation within UK Biobank can be found in Malcomson et al. [12], with a summary, including cut-offs and the data items used, provided in Additional file 1: Table S1. UK Biobank data on BMI and waist circumference, measured by trained research staff, were used to assess adherence to the body weight recommendation. BMI was calculated from body weight data, measured to the nearest 0.1 kg using the Tanita BC-418 MA body composition analyser, and height data, measured using a Seca 202 height measure. Waist circumference was measured at the natural indent (or umbilicus if the natural indent could not be located) using a Seca 200 tape measure. Self-reported data on time spent in moderate to vigorous physical activity (MVPA), collected using a short form of the International Physical Activity Questionnaire (IPAQ) [13], were used to assess adherence to the physical activity recommendation. Dietary data from the 24-h dietary assessment (the Oxford WebQ [14]) and from the short food frequency questionnaire (FFQ) were used to measure adherence to the remaining five recommendations on ‘Wholegrains, vegetables, fruits and beans’, ‘Fast-foods’, ‘Red and processed meat’, ‘Sugar-sweetened drinks’, and ‘Alcohol consumption’.

We assigned 0 points for non-adherence, 0.5 points for partial adherence, and 1 point for full adherence for each component of the score. For sub-components (BMI and waist circumference within ‘Healthy weight’, and fruits and vegetables and dietary fibre within ‘Wholegrains, vegetables, fruits and beans’), 0.25 points were given for partial adherence and 0.5 points for full adherence. Scores for all seven components were summed to yield a total score for each individual ranging from 0 to 7 points.

### Assessment of outcomes

The UK Biobank population has been linked electronically to population-based cancer registries (National Cancer Data Repository, Scottish Cancer Registry and Welsh Cancer Surveillance & Intelligence Unit) to identify prevalent and incident cancer cases (including information on cancer site and date of diagnosis). Completeness of case ascertainment in UK cancer registries is high (98–99%) [15, 16]. We used Cancer Registry data available until July 2019 for England and Wales and October 2015 for Scotland. Cancers were classified using the International Classification of Diseases, 10th revision (ICD-10). Our focus was on cancers diagnosed after UK Biobank recruitment and our outcomes were as follows: overall incident cancer (i.e. all cancers combined, C00-C97, excluding non-melanoma skin cancer (C44)) and 14 cancers for which lifestyle is an aetiological factor [2]: head and neck (C00–C14), oesophageal (C15), stomach (C16), colorectal (C18–C20), liver (C22), gallbladder (C23–24), pancreatic (C25), lung (C33–34), breast (C50), uterine (C54–C55), ovarian (C56), prostate (C61), kidney (C64–C65), and bladder (C67). We also considered subsites within the colorectum individually: namely, colon (C18.0), proximal colon (C18.0–18.4), distal colon (C18.5, C18.7), and rectum (C19–C20).

### Covariates

Data on sociodemographic factors, including sex and ethnicity, were self-reported and collected using a touchscreen questionnaire during the baseline assessment centre visit. Age was calculated from date of birth. Townsend Deprivation Index, an area-based measure of deprivation which accounts for unemployment, overcrowding, non-car ownership, and non-home ownership, was derived from each participant’s postcode at the time of study recruitment and was based on data from the preceding national census [17]. Smoking status was self-reported using a touchscreen questionnaire during the baseline assessment centre visit and categorised for analysis as ‘never’, ‘previous’ or ‘current’ smoker. As menopausal status was recorded at baseline only, to enable us to investigate associations for pre- and post-menopausal breast cancer separately, we estimated menopausal status by calculating the age at diagnosis or follow-up, as appropriate, and categorised women aged ≤ 50 years as pre-menopausal and those aged > 50 years as post-menopausal.

### Statistical analyses

Participants with missing data for the exposure of interest (adherence score), or any covariates, who completed less than two 24-h dietary assessments, and those with a prevalent cancer at baseline were excluded from the analysis (Additional file 1: Figure S1).

Cox-proportional hazard models were used to investigate associations between total adherence score and all cancers combined, as well as the 14 cancer sites and colorectal subsites individually. Each individual in the study population was followed over time from UK Biobank recruitment to cancer diagnosis or date of death (obtained through linkage to national death registries), or end of follow-up (July 2019 for England and Wales and October 2015 for Scotland), whichever occurred first. We conducted a landmark analysis to minimise the effect of reverse causation by excluding participants with new cancer cases in the first 2 years of follow-up. The total score was analysed, in the first instance, as a continuous variable (possible score range 0–7 points), estimating the hazard ratio (HR) associated with a 1-point score increment. It was also investigated as a categorical variable by dividing participants according to approximate score tertiles of the study population. Participants in the lowest score tertile (with lowest adherence to the 2018 Cancer Prevention Recommendations) were used as the reference group.

Model 1 was run for all cancer outcomes and included age, sex (if applicable), Townsend Deprivation Index and ethnicity as covariates. Model 2 included the covariates from model 1 plus smoking status. For female breast cancer, analyses were conducted overall and repeated after stratification according to estimated menopausal status at diagnosis, as described above. We repeated analyses after stratification according to sex and smoking status at baseline for all cancers.

Potential additional covariates, for example education, co-morbidities, family history of cancer (for prostate, breast, colorectal, and lung cancers), total energy intake, and those related to female reproductive cancers (e.g. parity, use of contraceptive pill or of hormone-replacement therapy), were tested by addition to Model 1 individually. None of the tested covariates changed the effect estimates by ≥ 5% and were therefore not added to the models. Nonetheless, in Additional file 1: Table S6, we present the findings for additional adjustment for mean total daily energy intake, multimorbidity, education, number of 24-h dietary assessments completed, and, for prostate, breast, lung, and colorectal cancers, family history of that cancer. For female cancers (breast, uterine, and ovarian), statistical models were additionally adjusted for menopausal status, use of oral contraceptives, use of hormone replacement therapy, age of menarche, age at first birth, and parity.

Cox-proportional hazard models were run in StataMP version 16 (Stata Corp, College Station, TX, USA). Results are presented as hazard ratios (HR) and 95% confidence intervals (95% CI). To allow for the possibility of non-linear relationships between adherence score and cancer risk, penalised cubic splines analyses were performed using R Statistical Software, version 3.6.0, with the package ‘survival’. For cancers for which there was a significant inverse association between total score and cancer incidence in the most fully adjusted model, we explored the range of total score compatible with the lowest achievable risk. Specifically, this was the range of total score where the lower 95% CI overlaps with the lowest HR. For all analyses, *P*-values < 0.05 (two-sided) were considered statistically significant. We also present the results using the Holm-Bonferroni method that accounts for multiple testing.

### Sensitivity analyses

We performed sensitivity analyses by deriving scores (i) with the inclusion of pure fruit juices within the sugar-sweetened drinks score component and (ii) using the original cut-points from the 2018 WCRF/AICR Score, based on US guidelines, to assess adherence to the recommendation to limit alcohol consumption [4] (described further in Additional file 1: Supplementary Methods). Our rationale for including pure fruit juices within a sensitivity analysis was two-fold. Firstly, the WCRF/AICR acknowledge that ‘natural fruit juice is a source of healthy nutrients but also contains a lot of sugar and has lost most of the fibre obtained by eating the whole fruit, so it is best not to drink more than one glass (150 ml) a day’ [2]. Furthermore, Shams-White et al. decided that sugar-sweetened beverages include ‘sugars present in honey, syrups, fruit juices, and fruit juice concentrate’ [4]. Secondly, in the UK Biobank population, fruit juice is the top contributor to energy intake (1.8% energy intake) and to intake of free sugars among the beverage subcategories [18]. Lastly, we performed a third sensitivity analysis for model 2 using the date of the last completed valid 24-h dietary assessment as the time of study entry.

## Results

### Participant characteristics

We included 94,778 UK Biobank participants (Additional file 1: Figure S1) and the participant characteristics are described in Table 1. During a median follow-up time of 7.9 (IQR 7.3–8.7) years, 7296 participants developed cancer. The three most common cancers were prostate (1818 cases), breast (1438 cases, of whom 1284 were in women who were post-menopausal, aged > 50 years at diagnosis) and colorectal (863 cases) cancers. Additional file 1: Table S2 compares the characteristics of those in the whole cohort vs. participants included in this analysis.

**Table 1:** UK Biobank participant characteristics according to approximate score tertiles of the study population

	**Low (0–3.5)**	**Middle (3.75–4.25)**	**High (4.5–7)**	**Overall (0–7)**
Total score (points)	2.84 (0.60)	4.00 (0.20)	5.01 (0.48)	3.84 (1.04)
Number of participants, *n* (%)	39,048 (41.2)	25,923 (27.4)	29,807 (31.5)	94,778 (100)
Sex,* n* (%)				
Females	16,476 (42.2)	14,187 (54.7)	19,965 (67.0)	50,628 (53.4)
Males	22,572 (57.8)	11,736 (45.3)	9842 (33.0)	44,150 (46.6)
Age (years)	55.7 (7.9)	56.1 (7.9)	55.8 (7.9)	55.8 (7.9)
Country of recruitment, *n* (%)				
England	35,648 (91.3)	23,781 (91.8)	27,348 (91.8)	86,777 (91.6)
Scotland	2113 (5.4)	1368 (5.3)	1604 (5.4)	5085 (5.4)
Wales	1287 (3.3)	774 (3.0)	855 (2.9)	2916 (3.1)
Education,* n* (%)				
College or University degree	16,959 (43.4)	12,755 (49.2)	16,092 (54.0)	45,806 (48.3)
A levels/AS levels or equivalent	5430 (13.9)	3590 (13.9)	3806 (12.8)	12,826 (13.53
O levels/GCSEs or equivalent	8293 (21.2)	4767 (18.4)	5077 (17.0)	18,137 (19.1)
CSEs or equivalent	1523 (3.9)	824 (3.2)	780 (2.6)	3127 (3.3)
NVQ or HND or HNC or equivalent	2330 (6.0)	1247 (4.8)	1104 (3.7)	4681 (4.9)
Other professional qualifications	1766 (4.5)	1242 (4.8)	1478 (5.0)	4486 (4.7)
None of the above	2670 (6.8)	1454 (5.6)	1403 (4.7)	5527 (5.8)
Do not know/prefer not to answer	77 (0.2)	44 (0.2)	67 (0.2)	188 (0.2)
Townsend deprivation index	− 1.66 (2.82)	− 1.77 (2.77)	− 1.61 (2.86)	− 1.67 (2.82)
Ethnicity, *n* (%)				
White	38,044 (97.4)	25,142 (97.0)	28,543 (95.8)	91,729 (96.8)
Mixed and other	373 (1.0)	277 (1.1)	422 (1.4)	1072 (1.1)
Asian or Asian British	271 (0.7)	262 (1.0)	435 (1.5)	968 (1.0)
Black or Black British	323 (0.8)	182 (0.7)	256 (0.9)	761 (0.8)
Chinese	37 (0.1)	60 (0.2)	151 (0.5)	248 (0.3)
Smoking, *n* (%)				
Never	21,301 (54.6)	15,286 (59.0)	18,391 (61.7)	54,978 (58.0)
Former smoker	14,363 (36.8)	8961 (34.6)	9945 (33.4)	33,269 (35.1)
Current smoker	3318 (8.5)	1642 (6.3)	1429 (4.8)	6389 (6.7)
Unknown	66 (0.2)	34 (0.1)	42 (0.1)	142 (0.2)

Participants with total score, full data for covariates in model 1, completed > 1 24-h dietary assessment and did not have cancer at baseline

Data are presented as means and standard deviation in brackets (SD) for total score, age and Townsend Deprivation Index. Data for sex, education, ethnicity and smoking status are presented as number of participants (*n*) and percentage in brackets (%)

### Adherence score and cancer incidence

**Fig. 1:**
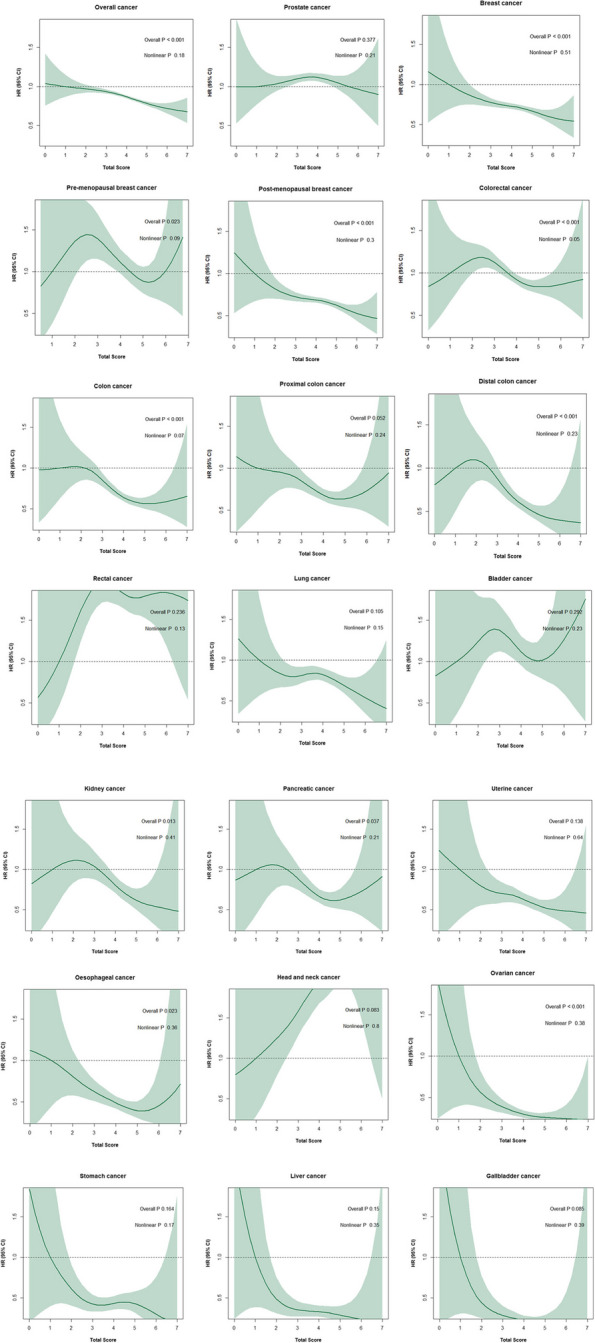
Associations between total score as a continuous variable and risk of all cancers combined and of cancers at individual anatomical sites. Hazard ratios (HR) with 95% confidence intervals (CI) and *p* values for fully adjusted statistical model (adjusted for age, sex (if applicable), Townsend deprivation index, ethnicity, and smoking status)

Figure 1 illustrates the associations between total adherence score and the incidence of all cancers combined and of individual cancer sites in the fully adjusted models. Cubic spline analyses suggested a non-linear relationship between adherence score and risk of colorectal cancer. For other cancers, there was no evidence that the relationship was non-linear.

When the score was assessed as a continuous variable, statistically significant associations were observed between the total score and risk of all cancers combined (HR per 1-unit increment in adherence score: 0.93 [95% CI: 0.90–0.95]), as well as breast (HR: 0.90 [95% CI: 0.86–0.95]), colorectal (HR: 0.90 [95% CI: 0.84–0.97]), kidney (HR: 0.82 [95% CI: 0.72–0.94]), oesophageal (HR: 0.84 [95% CI: 0.71–0.98]), ovarian (HR: 0.76 [95% CI: 0.65–0.90]), liver (HR: 0.78 [95% CI: 0.63–0.97]), and gallbladder (HR: 0.70 [95% CI: 0.53–0.93]) cancers in model 2 (Table 2). Adherence score was significantly associated with lung and pancreatic cancers for model 1, but associations were no longer significant after adjusting for smoking. For all cancers combined, and the sites for which there was a significant inverse association with total score, Additional file 1: Table S3 shows that the lowest risk was observed in those with a score between 5.75 and 7 points. This table also presents the range of total score compatible with the lowest achievable risk. When breast cancer was stratified according to menopausal status, the association with adherence score was significant for both pre-menopausal (aged ≤ 50 years at diagnosis) (HR: 0.83 [95% CI: 0.72–0.96]) and post-menopausal (aged > 50 years at diagnosis) (HR: 0.90 [95% CI: 0.85–0.95]) breast cancers. When associations with colorectal cancers were investigated at specific anatomical subsites, they were statistically significant for colon (HR: 0.83 [95% CI: 0.77–0.90]) and distal (HR: 0.75 [95% CI: 0.66–0.85]) cancers (Table 2).

**Table 2:** Associations between 1-point increment in total adherence score and risk of all cancers combined and of cancer at individual anatomical sites

			**Model 1**		**Model 2**	
**Cancer site**	**Total**	**Incident cancers**	**HR (95% CI)**	***P* value**	**HR (95% CI)**	***P* value**
All cancers combined	93,630	7296	**0.92 (0.90; 0.94)**	** < 0.001**^**a**^	**0.93 (0.90; 0.95)**	** < 0.001**^**a**^
Prostate	43,851	1818	1.01 (0.96; 1.05)	0.759	1.00 (0.96; 1.05)	0.959
Breast	50,337	1438	**0.90 (0.85; 0.95)**	** < 0.001**^**a**^	**0.90 (0.86; 0.95)**	** < 0.001**^**a**^
*Pre-menopausal*	1108	154	**0.83 (0.72; 0.96)**	**0.014**	**0.83 (0.72; 0.96)**	**0.013**
*Post-menopausal*	49,229	1284	**0.90 (0.85; 0.95)**	** < 0.001**^**a**^	**0.90 (0.85; 0.95)**	** < 0.001**^**a**^
Colorectal	94,656	863	**0.89 (0.83; 0.95)**	**0.001**^**a**^	**0.90 (0.84; 0.97)**	**0.003**^**a**^
*Colon*	94,698	575	**0.83 (0.76; 0.90)**	** < 0.001**^**a**^	**0.83 (0.77; 0.90)**	** < 0.001**^**a**^
*Distal*	94,734	242	**0.74 (0.65; 0.84)**	** < 0.001**^**a**^	**0.75 (0.66; 0.85)**	** < 0.001**^**a**^
*Proximal*	94,748	303	0.89 (0.80; 1.00)	0.050	0.90 (0.81; 1.01)	0.074
*Rectum*	94,734	342	1.00 (0.91; 1.12)	0.878	1.03 (0.93; 1.15)	0.588
Lung	94,760	431	**0.86 (0.78; 0.94)**	** < 0.001**^**a**^	0.93 (0.84; 1.02)	0.125
Kidney	94,754	221	**0.82 (0.72; 0.93)**	**0.003**^**a**^	**0.82 (0.72; 0.94)**	**0.004**
Pancreas	94,770	213	**0.87 (0.76; 0.99)**	**0.035**	0.88 (0.77; 1.00)	0.056
Uterus	50,588	212	0.93 (0.81; 1.06)	0.269	0.92 (0.81; 1.05)	0.227
Oesophagus	94,765	151	**0.81 (0.69; 0.95)**	**0.010**	**0.84 (0.71; 0.98)**	**0.031**
Ovary	50,603	147	**0.76 (0.65; 0.90)**	**0.001**^**a**^	**0.76 (0.65; 0.90)**	**0.001**^**a**^
Bladder	94, 756	141	0.88 (0.74; 1.03)	0.116	0.90 (0.76; 1.07)	0.240
Head and neck	94,763	129	1.17 (0.99; 1.40)	0.068	**1.21 (1.02; 1.44)**	**0.031**
Stomach	94,770	99	0.97 (0.79; 1.18)	0.725	0.98 (0.80; 1.19)	0.830
Liver	94,773	82	**0.77 (0.62; 0.96)**	**0.017**	**0.78 (0.63; 0.97)**	**0.024**
Gallbladder	94,777	49	**0.71 (0.54; 0.94)**	**0.017**	**0.70 (0.53; 0.93)**	**0.013**

Data are presented as hazard ratio (HR) with 95% confidence intervals in parentheses (95% CIs) per 1-point increment in score. Model 1 was adjusted for age, sex, Townsend deprivation index, and ethnicity, and Model 2 was additionally adjusted for smoking status

^a^Following correction using the Holm-Bonferroni method *P* < 0.05

Following correction with the Holm-Bonferroni method for multiple testing, findings for associations between the score and risk of all cancers combined, breast cancer (including post-menopausal breast cancer), colorectal cancer (including colon and distal colon cancers), and ovarian cancer remained statistically significant in model 2.

When analyses were repeated for approximate score tertiles of the study population, compared with participants in the lowest tertile (scoring 0–3.5 points), based on the most fully-adjusted models, those in the highest tertile (scoring 4.5–7 points) had reduced risk of all cancers combined (HR 0.84 (95% CI: 0.79–0.89) and of breast (HR 0.82 [95% CI: 0.73–0.93]), colorectal (HR 0.79 [95% CI: 0.66–0.93]), kidney (HR 0.64 [95% CI: 0.45–0.92]), oesophageal (HR 0.64 [95% CI: 0.41–0.99]), and ovarian (HR 0.57 [95% CI: 0.39–0.84]) cancers (Table 3). Participants in the middle tertile, with scores between 3.75 and 4.25 points, had lower risk of all cancers combined (HR 0.92 [95% CI: 0.87–0.97]) and of breast (HR 0.82 [95% CI: 0.72–0.94]) and colorectal (HR 0.79 [95% CI: 0.67–0.93]) cancer, compared with those in the lowest adherence group. When breast cancer was stratified by menopausal status, the association with adherence score tertile was significant for both pre- (HR for highest vs. lowest score tertile: 0.67 [95% CI: 0.47–0.96]) and post-menopausal (HR 0.82 [95% CI: 0.72–0.93]) women. For colorectal subsites, significant associations were seen for colon and distal cancers, but not proximal or rectal cancers. Following correction with the Holm-Bonferroni method for multiple testing, differences in the risk of all cancers combined and of breast (including those in post-menopausal women), colon, and distal colon cancers remained statistically significant. Differences between the middle and lowest score tertile remained statistically significant in model 2 for colon cancer only.

**Table 3:** Associations between total adherence score, categorised according to approximate score tertiles of the study population, and risk of all cancers combined and of cancers at individual anatomical sites

			**Low score (0–3.5)**	**Mid score (3.75–4.25)**				**Higher score (4.5–7)**			
				**Model 1**		**Model 2**		**Model 1**		**Model 2**	
**Cancer site**	**Total**	**Incident cancers**	**HR (95% CI)**	**HR (95% CI)**	***P* value**	**HR (95% CI)**	***P* value**	**HR (95% CI)**	***P* value**	**HR (95% CI)**	***P* value**
All cancers combined	93,630	7296	1.00 (ref)	**0.91 (0.86; 0.97)**	**0.001**^**a**^	**0.92 (0.87; 0.97)**	**0.004**	**0.83 (0.78; 0.87)**	** < 0.001**^**a**^	**0.84 (0.79; 0.89)**	** < 0.001**^**a**^
Prostate	43,851	1818	1.00 (ref)	1.06 (0.95; 1.18)	0.275	1.06 (0.95; 1.18)	0.306	0.98 (0.87; 1.10)	0.686	0.96 (0.85; 1.08)	0.499
Breast	50,337	1438	1.00 (ref)	**0.82 (0.72; 0.93)**	**0.003**	**0.82 (0.72; 0.94)**	**0.003**	**0.82 (0.73; 0.93)**	**0.001**^**a**^	**0.82 (0.73; 0.93)**	**0.002**^**a**^
*Pre-menopausal*	1108	154	1.00 (ref)	**0.58 (0.38; 0.89)**	**0.013**	**0.58 (0.38; 0.89)**	**0.013**	**0.67 (0.47; 0.97)**	**0.032**	**0.67 (0.47; 0.96)**	**0.031**
*Post-menopausal*	49,229	1284	1.00 (ref)	**0.83 (0.72; 0.96)**	**0.010**	**0.83 (0.72; 0.96)**	**0.011**	**0.81 (0.72; 0.93)**	**0.002**^**a**^	**0.82 (0.72; 0.93)**	**0.002**^**a**^
Colorectal	94,656	862	1.00 (ref)	**0.78 (0.66; 0.92)**	**0.004**	**0.79 (0.67; 0.93)**	**0.006**	**0.76 (0.65; 0.90)**	**0.001**^**a**^	**0.79 (0.66; 0.93)**	**0.005**
*Colon*	94,698	575	1.00 (ref)	**0.69 (0.56; 0.85)**	** < 0.001**^**a**^	**0.69 (0.57; 0.85)**	**0.001**^**a**^	**0.67 (0.55; 0.82)**	** < 0.001**^**a**^	**0.69 (0.56; 0.84)**	** < 0.001**^**a**^
*Distal*	94,734	242	1.00 (ref)	**0.62 (0.46; 0.86)**	**0.003**	**0.62 (0.45; 0.85)**	**0.003**	**0.54 (0.39; 0.76)**	** < 0.001**^**a**^	**0.56 (0.40; 0.77)**	** < 0.001**^**a**^
*Proximal*	94,748	303	1.00 (ref)	0.77 (0.58; 1.02)	0.069	0.78 (0.59; 1.03)	0.084	**0.75 (0.57; 1.00)**	**0.057**	0.77 (0.58; 1.02)	0.067
*Rectum*	94,734	342	1.00 (ref)	0.97 (0.74; 1.25)	0.789	0.99 (0.76; 1.28)	0.943	0.97 (0.74; 1.26)	0.798	1.01 (0.78; 1.32)	0.926
Lung	94,760	431	1.00 (ref)	0.92 (0.73; 1.15)	0.465	1.02 (0.81; 1.27)	0.895	**0.67 (0.52; 0.85)**	**0.001**^**a**^	0.80 (0.63; 1.02)	0.073
Kidney	94,754	221	1.00 (ref)	0.85 (0.62; 1.16)	0.299	0.85 (0.62; 1.17)	0.322	**0.64 (0.45; 0.90)**	**0.011**	**0.64 (0.45; 0.92)**	**0.014**
Pancreas	94,770	213	1.00 (ref)	0.72 (0.52; 1.01)	0.061	0.74 (0.53; 1.04)	0.076	0.73 (0.52; 1.01)	0.058	0.75 (0.53; 1.04)	0.087
Uterus	50,588	212	1.00 (ref)	1.00 (0.71; 1.40)	0.994	0.99 (0.71; 1.39)	0.964	0.84 (0.61; 1.16)	0.287	0.83 (0.60; 1.14)	0.250
Oesophagus	94,765	151	1.00 (ref)	0.89 (0.61; 1.29)	0.534	0.92 (0.63; 1.34)	0.665	**0.60 (0.39; 0.92)**	**0.020**	**0.64 (0.41; 0.99)**	**0.046**
Ovary	50,603	147	1.00 (ref)	0.69 (0.46; 1.03)	0.071	0.69 (0.46; 1.03)	0.070	**0.57 (0.39; 0.84)**	**0.005**	**0.57 (0.39; 0.84)**	**0.005**
Bladder	94,756	141	1.00 (ref)	0.70 (0.47; 1.06)	0.093	0.73 (0.48; 1.10)	0.135	0.67 (0.43; 1.03)	0.066	0.72 (0.47; 1.11)	0.137
Head and Neck	94,763	129	1.00 (ref)	1.45 (0.95; 2.22)	0.086	1.51 (0.98; 2.31)	0.060	1.42 (0.93; 2.18)	0.108	1.52 (0.99; 2.33)	0.057
Stomach	94,770	99	1.00 (ref)	1.01 (0.63; 1.62)	0.982	1.02 (0.64; 1.65)	0.920	0.94 (0.57; 1.54)	0.804	0.97 (0.59; 1.59)	0.899
Liver	94,773	82	1.00 (ref)	0.78 (0.46; 1.33)	0.364	0.80 (0.47; 1.35)	0.397	0.66 (0.38; 1.15)	0.142	0.68 (0.39; 1.18)	0.172
Gallbladder	94,777	49	1.00 (ref)	0.68 (0.34; 1.35)	0.269	0.67 (0.34; 1.33)	0.251	0.56 (0.27; 1.15)	0.115	0.54 (0.26; 1.12)	0.098

Data are presented as hazard ratio (HR) with 95% confidence intervals in parentheses (95% CIs). Lowest score tertile was the reference group. Model 1 was adjusted for age, sex, Townsend deprivation index, and ethnicity. Model 2 was additionally adjusted for smoking status

^a^Following correction using the Holm-Bonferroni method *P* < 0.05

When stratifying according to sex, inverse associations between score and risk of colorectal, lung, pancreatic, oesophageal, liver, and gallbladder cancers were statistically significant for male participants only (Additional file 1: Table S4). When repeating analyses stratified according to smoking status at baseline, the observed associations between score and risk of all cancers combined and of breast cancer were significant in never or former, but not current, smokers (Additional file 1: Table S5). Associations between score and risk of colorectal, kidney, and ovarian cancers were significant in participants who had never smoked, and in former smokers for lung (highest vs. lowest score tertile only) and pancreatic cancers (Additional file 1: Table S5). Further, inverse associations between adherence score when analysed as a continuous variable (per 1-point increment in score) and oesophageal cancer were significant in current smokers (Additional file 1: Table S5). We additionally adjusted for mean total daily energy intake, multimorbidity, education, number of 24-h dietary assessments completed, and for prostate, breast, lung, and colorectal cancers, family history of that cancer. For female cancers (breast, uterine, and ovarian), statistical models were additionally adjusted for menopausal status, use of oral contraceptives, use of hormone replacement therapy, age of menarche, age at first birth, and parity. The findings from these additional analyses were comparable with those in the original fully adjusted model (model 2), with the exception of associations between adherence score and risk of oesophageal, ovarian, and liver (score tertiles) cancers where the same patterns of association were observed but the risk estimates were somewhat attenuated and no longer statistically significant (Additional file 1: Table S6).

### Sensitivity analyses

The mean total scores when (i) including pure fruit juices within the sugar-sweetened drinks score component and (ii) using the original score cut-points for the alcohol score component (based on US guidelines) were 3.62 (SD 1.04) and 3.88 (SD 1.03) points, respectively. Sensitivity analyses including pure fruit juices within the sugar-sweetened drinks score component produced findings that were generally consistent with the primary analyses (Additional file 1: Table S7 and S8). Exceptions were seen for proximal colon, oesophageal, and head and neck cancer. For proximal colon cancer, significant inverse associations were observed between total score and risk when the score was treated both as a continuous variable and in tertiles; associations with oesophageal cancer were no longer statistically significant when total score was considered as a continuous variable; and participants in the highest score tertile had a significantly raised risk of head and neck cancer.

In sensitivity analyses based on US alcohol cut-offs, results were largely unchanged from the primary analysis, except for proximal colon cancer. In contrast to the primary analysis, risk for this cancer was statistically significantly lower in the highest *versus* lowest score tertile in this sensitivity analysis (Additional file 1: Table S9 and S10). Lastly, our results were similar when we ran a sensitivity analysis setting the date of the last completed valid 24-h dietary assessment as the time of study entry (Additional file 1: Table S11), with the exception of pre-menopausal breast cancers when analysing the score as a continuous variable which became borderline significant (*p* = 0.055). Further, in addition to the described associations, we found an additional inverse association between total score and the risk of pancreatic cancer (HR per 1-point increment: 0.81 [95% CI: 0.70; 0.92], *p* = 0.002, and HR for highest vs. lowest score tertile: 0.60 [95% CI: 0.42–0.84], *p* = 0.003).

## Discussion

This is the first study to investigate associations between cancer risk in a UK cohort and adherence to the 2018 WCRF/AICR Cancer Prevention Recommendations assessed using a fully operationalised version of the standardised scoring system [4]. Further, it is the first to investigate associations between 2018 WCRF/AICR Score and the risk of all lifestyle-related cancers individually. To date, internationally, associations between adherence to the latest, 2018 version of the WCRF/AICR Cancer Prevention Recommendations (derived using various scoring systems including the 2018 WCRF/AICR Score) and cancer risk have been restricted to studies of prostate, breast, colorectal, lung, pancreatic, and uterine cancers and chronic lymphocytic leukaemia. Thus, we are the first to investigate associations between the 2018 WCRF/AICR Score and risks of kidney, bladder, ovarian, head and neck, oesophageal, stomach, liver and gallbladder cancers individually. Findings from studies that have investigated associations between the earlier 2007 version of the Cancer Prevention Recommendations and the incidence of multiple cancer sites have been summarised by Solans et al. [3].

### All cancers combined

We found a significant inverse association between total adherence score and risk of all cancers combined equivalent to a 7% reduction in risk per 1-point increment in score when adjusting for age, sex, deprivation, ethnicity, and smoking status. Further, compared with those in the lowest score tertile (0–3.5 points), participants in the middle (3.75–4.25 points) and highest (4.5–7 points) score tertiles had an 8% and 16% lower risk of developing all cancers combined, respectively.

To our knowledge, only one other study [11] has investigated associations between adherence to the 2018 Cancer Prevention Recommendations and the risk of combined cancers overall using the 2018 WCRF/AICR Score. In the Swedish Mammograph Cohort and Cohort of Swedish Men which included 12,693 incident cancer cases over a 15-year follow-up, Kaluza and colleagues reported a 3% reduction in cancer risk per 1-point increment in score [11], which is considerably less than the 7% reduction observed in the present study. In addition, these authors reported a 12% lower cancer risk in participants in the highest score category (> 4 points) compared with those in the lowest category (0–2 points) [11]. This emphasises the need to examine these associations in different populations worldwide.

### Breast cancer

We observed a 10% reduction in the risk of breast cancer per 1-unit increment in adherence score. The lowest risk of breast cancer was observed in participants with a total score between 5.75 and 7 points, corresponding to a HR between 0.54 and 0.60. Further, women in the highest score tertile (≥ 4.5 points) had an 18% lower risk of developing breast cancer compared with those scoring ≤ 3.5 points. To our knowledge, only three other studies have fully applied the standardised scoring system to assess associations between adherence to the 2018 Cancer Prevention Recommendations and breast cancer risk [5, 10, 19]. In the US NIH-AARP Diet and Health Study, which included post-menopausal women only, Korn and colleagues reported a reduction in risk per 1-point increase in score of 7–19%, depending on smoking status [10]. In a Spanish study of 10,930 women (including 119 incident breast cancers) with a median follow-up of 12 years, a non-significant trend for a lower risk of breast cancer in women with higher adherence scores was reported [5]. However, there was a 73% lower risk of post-menopausal breast cancer in women with maximum compliance (scoring > 5 points) compared with those with minimal compliance (scoring ≤ 3 points) [5]. In the South African Breast Cancer case–control study, Jacobs and colleagues reported a 46% lower risk of breast cancer in women with greater adherence (> 4.5 *versus* < 3.25 points) in post-menopausal, but not pre-menopausal, women [19]. Note that the latter study operationalised the eighth, optional component of the 2018 WCRF/AICR score regarding breastfeeding.

Two studies have used UK Biobank data to examine lifestyle and breast cancer risk but without assessing adherence to the Cancer Prevention Recommendations using the 2018 WCRF/AICR Score. One study explored associations between a modified ‘Healthy Lifestyle Index’ score, which included components of the scoring system, and breast cancer risk in 146,326 women [20]. Specifically, the score included physical activity, BMI, waist circumference, smoking, and the intake of fruits and vegetables, cereals and grains, and red and processed meat. Women in the highest healthy lifestyle score tertile had 22% and 31% lower risks of pre-menopausal and post-menopausal breast cancer, respectively. A further study calculated a ‘lifestyle score’, ranging from 0 to 6 points, and found an 8% reduction in risk of invasive breast cancer per 1-point increment in score [21]. However, these authors used dietary data collected using the baseline touchscreen questionnaire only, which does not allow for the assessment of the intake of UPFs, estimated ‘partial fibre’ intake only, and used a question on sugar avoidance as a proxy to assess adherence to the recommendation to ‘limit sugary drinks’ [21].

### Colorectal cancer

We observed a 10% reduction in colorectal cancer risk per 1-point increment in score. Furthermore, participants in the middle and highest score tertiles had a 21% lower risk of developing colorectal cancer compared with those in the lowest score tertile. To our knowledge, only one other study, conducted in the USA, has assessed relationships between the risk of colorectal cancer and adherence to the Cancer Prevention Recommendations by fully operationalising the 2018 WCRF/AICR Score [10]. In participants who had never smoked, Korn and colleagues reported a 13% and 10% reduction in risk of colorectal cancer per 1-point increment in score in males and females, respectively [10]. Significant associations of a similar magnitude were also observed in former, but not current, smokers [10]. In the present study, following stratification according to baseline smoking status, associations between score and colorectal cancer risk were only statistically significant in UK Biobank participants who reported never smoking.

Four studies have investigated adherence to the 2018 WCRF/AICR Cancer Prevention Recommendations and colorectal cancer risk by devising their own scoring systems [6, 22–24]. Using data from the Spanish PREvencion con DIeta MEDiterranea (PREDIMED) cohort, Barrubes et al. assessed associations between adherence to the Cancer Prevention Recommendations in 7216 older men and women at increased cardiovascular risk [6]. This analysis used an alternative scoring system with different cut-offs from those proposed by Shams-White and colleagues [4]; for example, the researchers did not operationalise the waist circumference sub-component but instead assessed weight gain throughout adulthood using tertiles as cut-offs [6]. During a median follow-up time of 6 years, during which 97 colorectal cancer cases were identified, there was a 21% reduction in colorectal cancer risk per 1-point increment in adherence score. In the Nurses’ Health Study and the Health Professionals Follow-Up Study, Petimar and colleagues reported a 36% and 14% lower risk of colorectal cancer in men and women, respectively, in the highest versus the lowest adherence score quintile [22]. Similarly, in the other two studies which were conducted in the USA [23] and Morocco [24], greater adherence to the Cancer Prevention Recommendations was associated with lower colorectal cancer risk [24, 25]. The consistency of the patterns of relationships between adherence and colorectal cancer risk in different populations indicates the importance of public health messages about a healthy lifestyle—rather than individual aspects of lifestyle—for prevention of this cancer.

### Risk of other cancers

To our knowledge, we are the first to report lower risks of kidney, oesophageal, ovarian, liver, and gallbladder cancers with greater adherence to the 2018 WCRF/AICR Cancer Prevention Recommendations. In the EPIC Study, Romaguera et al. reported 42% lower risk of oesophageal cancer, 29% lower risk of kidney cancer, and 15% lower risk of liver cancer in participants in the fourth and fifth highest categories (≥ 4 points) of a score used to assess adherence to the previous (2007) version of the WCRF/AICR Cancer Prevention Recommendations compared with those in the lowest score category (0–2 and 0–3 points for men and women, respectively) [25]. The WCRF/AICR have concluded that there is strong evidence for increased risk of oesophageal adenocarcinoma and of kidney, gallbladder, liver, and ovarian cancers with greater body fatness and for increased risk of liver cancer and of oesophageal squamous cell carcinoma with higher alcohol intake [2]. The evidence for the influence of additional lifestyle factors on these cancers is limited—for example, the WCRF/AICR report limited evidence for a protective effect of physical activity against liver and oesophageal cancers [2]. Because of the potential for cancer prevention, these findings warrant further investigation and confirmation in other populations.

Since mortality from pancreatic cancer is high and there is little possibility of secondary or tertiary prevention, studies which identify the role of potentially modifiable risk factors are important in terms of primary prevention. In a prospective cohort study in the USA, Zhang et al. observed a 33% lower pancreatic cancer risk in participants scoring ≥ 5 points on the adherence score compared with those scoring < 4 points, and a 14% reduction in pancreatic cancer risk per 1-point increment in score [9]. These findings are similar to those observed in our study (13% reduction in pancreatic cancer risk per 1-point increment in score). However, after adjusting for smoking status the association was only borderline significant (*p* = 0.056) in our study. Importantly, when we performed a sensitivity analysis using the date of the last completed valid 24-h dietary assessment as the time of study entry, we observed a 19% lower risk in pancreatic cancer per 1-point increment in score. The American study was of a similar size to the present study (95,962 participants) but included more pancreatic cancer cases (337 *versus* 213). Furthermore, although they assessed adherence using the 2018 WCRF/AICR Score, the authors included the optional eight component regarding breastfeeding (score range 0–8 points *versus* 0–7 points in the present study). The importance of breastfeeding in influencing pancreatic cancer risk is uncertain. The Norwegian Women and Cancer study reported an inverse linear relationship between cumulative breastfeeding duration and pancreatic cancer incidence [26], although no significant associations were found in a study in Japan [27].

The Spanish population-based case–control CAPLIFE study, which also fully operationalised the 2018 WCRF/AICR Score, reported a 19% reduction in risk of prostate cancer per 1-point increase in score, but there were no significant associations when investigating associations according to score tertiles [7]. We did not find associations between adherence score and prostate cancer incidence in the present study, despite including 1818 prostate cancer cases. Similarly, in the NIH-AARP Diet and Health Study which included 920 prostate cancer cases, adherence scores were not significantly associated with prostate cancer risk regardless of smoking status [10]. The evidence for associations between lifestyle and prostate cancer is limited, with evidence only for body fatness and risk of advanced prostate cancer.

Lastly, we were somewhat surprised by the increased risk of head and neck cancers with greater adherence score, although this was limited to analyses on the score as a continuous variable, after adjusting for smoking status. The explanation for this is not immediately obvious. It may be due to the fact that there are multiple (sub)sites within head and neck cancers which vary in their aetiology; in particular, cancers at some subsites are driven largely by exposure to human papillomavirus while others are heavily influenced by tobacco and alcohol exposure. Further analyses examining adherence and risk of cancers at different head and neck (sub)sites are warranted. Only one study has reported a reduced risk of oral cavity and pharyngeal cancers and laryngeal cancer with greater adherence to the previous (2007) version of the Cancer Prevention Recommendations using data from two Italian case–control studies [28].

### Strengths and limitations

We are one of the few studies to fully operationalise the 2018 WCRF/AICR Score to assess adherence to the 2018 WCRF/AICR Cancer Prevention Recommendations [4, 29], allowing for comparability across studies, and we are the first to do so for a UK-based prospective cohort study. Moreover, as encouraged by the score creators, we applied national cut-offs to assess adherence to the recommendation to limit alcohol consumption. In addition, we ran sensitivity analyses using a score derived with the original cut-offs to assess adherence to the alcohol recommendation, based on US guidelines. More generally, we conducted a series of sensitivity analyses around the multivariable models; the results of these largely followed a similar pattern as to the primary analyses, suggesting our findings are robust.

A limitation of our study is that the UK Biobank cohort is not fully representative of the general population in the UK; participants were older, more often female and less socioeconomically deprived, and had ‘healthier’ lifestyles (less likely to have obesity and to smoke, and consumed less alcohol) [30]. Although mean adherence score for participants in the UK Biobank may be higher than that for the general population, our findings on comparisons of different levels of adherence should be generalisable. Further, the characteristics of the UK Biobank participants with sufficient data to allow us to derive a total score, included in the present study, are broadly similar to the rest of the UK Biobank participants, including the proportions of males and females and smoking status [31]. Nonetheless, we adjusted for these as potential confounding factors in our analyses. We carefully assessed the covariates to be included in our statistical models, by testing potential confounders to be added to the statistical models individually, as also described by van Zutphen and colleagues [32]. Nonetheless, we ran an additional model which included potential confounders such as education, multimorbidity, and for female cancers, female-related factors such as contraceptive use, and found that our results were largely unchanged with the exception of associations between the score and risk of oesophageal, ovarian, and liver cancers which were somewhat attenuated and no longer reached statistical significance. As with any such study, our analyses may be subject to residual or unmeasured confounders. We did not include cancer screening as a potential confounder as this variable is likely to be of questionable quality and to have different meanings for different cancers at different times. For example, the screening programmes started at different times (colorectal cancer screening in England began for people aged 60–69 in 2006), and analysis of the first 2.6 million invitations to participate in England shows that uptake was only 54% and that there were considerable inequalities in uptake in ethnically diverse areas and a striking gradient by socioeconomic status [33]. Moreover, whether self-report of having been screened is likely to have a direct influence on adherence is unclear. Lastly, a limitation of the data on smoking status is that this was self-reported at baseline and does not provide information on smoking intensity or the timing of when former smokers quit.

We applied robust methodology to operationalise the 2018 WCRF/AICR Score and to define the participants to be included in our study, including only participants who had completed at least two 24-h dietary assessments. However, this meant that we significantly reduced the cohort size. One of the consequences of this was that, for some cancers, we do not have a large number of cases, limiting our statistical power and resulting in wide CIs. Nonetheless, we are the first to investigate associations between adherence score and the risk of less common cancers including ovarian, stomach, and liver cancers. Further, we conducted a landmark analysis to minimise the effect of reverse causation by excluding cancer cases in the first 2 years of follow-up. However, the results should be interpreted with caution as, although we investigated associations with multiple cancers and have conducted a range of sensitivity analyses, we did not choose a priori to adjust for multiple testing [34].

The dietary and physical activity data used to operationalise the 2018 WCRF/AICR Score were collected using self-reported questionnaires, which may be prone to misreporting. However, estimates of energy and nutrient intake obtained from the Oxford WebQ online 24-h dietary questionnaire that was used to collect dietary data in UK Biobank correlated well with objective biomarkers for protein, potassium, and total sugar intake and total energy expenditure estimated by accelerometry [35]. Likewise, self-report physical activity data showed similar relationships with morbidity and mortality outcomes in UK Biobank to those observed using objective measures of physical activity [36]. Estimating habitual dietary intake is challenging and there is no ‘gold standard’ methodology. We have adopted an approach that is used widely in epidemiological studies to estimate habitual dietary intake using means of the completed 24-h dietary assessments, and included only participants who completed at least two Oxford WebQs—the latter criterion was applied because a single 24-h dietary assessment is less likely to represent long-term (habitual) dietary intake. In the UK Biobank, Carter and colleagues have concluded that taking the mean of at least two 24-h dietary assessments may be similar to FFQs for capturing longer-term dietary intakes [37], and Bradbury and colleagues have reported that dietary intake stayed relatively stable during 4 years of follow-up [38]. Further, the Oxford WebQ used for the 24-h dietary assessments has been recently validated and is considered to perform well in estimating dietary intake [35, 39]. In addition, when we performed a sensitivity analysis setting the date of the last completed valid 24-h dietary assessment as the time of study entry (to address potential concerns around immortal time bias), associations between the score, both as a continuous variable and as score tertiles, and cancer incidence were similar in terms of pattern, magnitude effect estimates, and statistical significance; in addition, a significant inverse association between total score and risk of pancreatic cancer was observed. It should be noted that UK Biobank participants who completed at least one 24-h dietary assessment were more likely to be women, older, of white ethnic background, less deprived, and more educated compared with non-responders, and those who completed multiple assessments were also more likely to be White, older, and more highly educated compared with those who only complete one [40].

## Conclusions

In conclusion, in the UK Biobank cohort, greater adherence to the 2018 WCRF/AICR Cancer Prevention Recommendations, assessed using a standardised scoring system, was associated with significantly reduced risk of all cancers combined and of breast, colorectal, kidney, oesophageal, ovarian, liver, and gallbladder cancers. Our findings support promoting compliance with the 2018 WCRF/AICR Cancer Prevention Recommendations for cancer prevention in the UK. Further studies are required to confirm the novel findings for kidney, oesophageal, ovarian, liver, and gallbladder cancers, in particular, in other populations, and we encourage researchers to fully operationalise the 2018 WCRF/AICR Score to facilitate comparability across studies. Further research to better understand which recommendations are driving the associations observed with cancer risk, as well as to explore the weightings allocated to the individual components (currently all seven or eight components are of equal weighting) would be of value.

### Supplementary Information

**Additional file 1: Supplementary Methods. **Calculation of alternative scores for sensitivity analyses.** Figure S1. **Flow chart of UK Biobank Study participants included in the present study.** Table S1. **Operationalisation of ‘2018 WCRF/AICR Score’ to assess adherence to the 2018 WCRF/AICR Cancer Prevention Recommendations, as devised by Shams-White et al. (2019).** Table S2. **Sociodemographic characteristics of UK Biobank participants with a total adherence score, who are included in the present analysis, and those without a total score, who were excluded. **Table S3. **Total score, lower and upper limits, and corresponding hazard ratios (HR) associated with lowest observed cancer risk for those cancers for which a significant inverse association with total score was observed.** Table S4. **Associations between total adherence score and risk of all cancers combined and of cancer at individual anatomical sites, stratified according to sex. **Table S5. **Associations between total adherence score and risk of all cancers combined and of cancer at individual anatomical sites, stratified according to smoking status.** Table S6.  **Associations between total adherence score and risk of all cancers combined and of cancer at individual anatomical sites, adjusting for additional confounders. **Table S7. **Sensitivity analysis for associations between 1-point increment in total score including fruit juices for the sugar-sweetened drinks score component and risk of all cancers and of cancer at individual anatomical sites.** Table S8. **Sensitivity analysis for associations between total score including fruit juices for the sugar-sweetened drinks score component, categorised according to score tertiles, and risk of all cancers and of cancers at individual anatomical sites. **Table S9. **Sensitivity analysis for associations between 1-point increment in total score using original cut-points (based on US guidelines) for alcohol score component and risk of all cancers and of cancer at individual anatomical sites. **Table S10. **Sensitivity analysis for associations between total score using original cut-points (based on US guidelines) for alcohol score component, categorised according to score tertiles, and risk of all cancers and of cancers at individual anatomical sites. **Table S11. **Sensitivity analysis for associations between total score and risk of all cancers and of cancers at individual anatomical sites using the date of completion of last 24-hour dietary assessment as baseline

## Data Availability

Data are available upon request from UK Biobank (www.ukbiobank.ac.uk).

## Abbreviations

AICR: American Institute for Cancer Research
aUPF: Adapted ultra-processed foods
BMI: Body mass index
FFQ: Food frequency questionnaire
HR: Hazard ratio
IPAQ: International Physical Activity Questionnaire
IQR: Interquartile range
MET: Metabolic-equivalent of task
MVPA: Moderate to vigorous physical activity
NDNS: National Diet and Nutrition Survey
PA: Physical activity
UPF: Ultra-processed food
WCRF: World Cancer Research Fund
95% CI: 95% Confidence interval

## References

[1] Brown KF, Rumgay H, Dunlop C, Ryan M, Quartly F, Cox A (2018). The fraction of cancer attributable to modifiable risk factors in England, Wales, Scotland, Northern Ireland, and the United Kingdom in 2015. Br J Cancer.

[2] WCRF/AICR. World Cancer Research Fund/American Institute for Cancer Research Diet, Nutrition, Physical Activity and Cancer: A Global Perspective. Continuous Update Project Expert Report 2018. 2018.

[3] Solans M, Chan DSM, Mitrou P, Norat T, Romaguera D (2020). A systematic review and meta-analysis of the 2007 WCRF/AICR score in relation to cancer-related health outcomes. Ann Oncol.

[4] Shams-White MM, Brockton NT, Mitrou P, Romaguera D, Brown S, Bender A, et al. Operationalizing the 2018 World Cancer Research Fund/American Institute for Cancer Research (WCRF/AICR) Cancer Prevention Recommendations: A Standardized Scoring System. Nutrients. 2019;11(7):1572. 10.3390/nu11071572 PMC6682977 31336836

[5] Barrios-Rodriguez R, Toledo E, Martinez-Gonzalez MA, Aguilera-Buenosvinos I, Romanos-Nanclares A, Jimenez-Moleon JJ. Adherence to the 2018 World Cancer Research Fund/American Institute for Cancer Research Recommendations and Breast Cancer in the SUN Project. Nutrients. 2020;12(7):2076. 10.3390/nu12072076 PMC7400833 32668662

[6] Barrubés L, Babio N, Hernandez-Alonso P, Toledo E, Ramirez Sabio JB, Estruch R, et al. Association between the 2018 WCRF/AICR and the low-risk lifestyle scores with colorectal cancer risk in the Predimed study. J Clin Med. 2020;9(4):1215. 10.3390/jcm9041215 PMC7230705 32340309

[7] Olmedo-Requena R, Lozano-Lorca M, Salcedo-Bellido I, Jimenez-Pacheco A, Vazquez-Alonso F, Garcia-Caballos M, et al. Compliance with the 2018 World Cancer Research Fund/American Institute for Cancer Research Cancer Prevention Recommendations and Prostate Cancer. Nutrients. 2020;12(3):768. 10.3390/nu12030768 PMC7146507 32183345

[8] Malcomson FC, Wiggins C, Parra-Soto S, Ho FK, Celis-Morales C, Sharp L, et al. Adherence to the 2018 World Cancer Research Fund (WCRF)/ American Institute for Cancer Research (AICR) Cancer Prevention Recommendations and cancer risk: a systematic review and meta-analysis. Cancer. 2023;129(17):2655–70. 10.1002/cncr.34842 37309215

[9] Zhang ZQ, Li QJ, Hao FB, Wu YQ, Liu S, Zhong GC (2020). Adherence to the 2018 World Cancer Research Fund/American Institute for Cancer Research cancer prevention recommendations and pancreatic cancer incidence and mortality: A prospective cohort study. Cancer Med.

[10] Korn AR, Reedy J, Brockton NT, Kahle LL, Mitrou P, Shams-White MM (2022). The 2018 World Cancer Research Fund/American Institute for Cancer Research Score and Cancer Risk: A Longitudinal Analysis in the NIH-AARP Diet and Health Study. Cancer Epidemiol Biomarkers Prev.

[11] Kaluza J, Harris HR, Hakansson N, Wolk A (2020). Adherence to the WCRF/AICR 2018 recommendations for cancer prevention and risk of cancer: prospective cohort studies of men and women. Br J Cancer.

[12] Malcomson FC, Parra-Soto S, Lu L, Ho FK, Perez-Cornago A, Shams-White MM (2023). Operationalisation of a standardised scoring system to assess adherence to the World Cancer Research Fund/American Institute for Cancer Research cancer prevention recommendations in the UK biobank. Front Nutr.

[13] Craig CL, Marshall AL, Sjostrom M, Bauman AE, Booth ML, Ainsworth BE (2003). International physical activity questionnaire: 12-country reliability and validity. Med Sci Sports Exerc.

[14] Liu B, Young H, Crowe FL, Benson VS, Spencer EA, Key TJ (2011). Development and evaluation of the Oxford WebQ, a low-cost, web-based method for assessment of previous 24 h dietary intakes in large-scale prospective studies. Public Health Nutr.

[15] Brewster DH, Stockton DL (2008). Ascertainment of breast cancer by the Scottish Cancer Registry: an assessment based on comparison with five independent breast cancer trials databases. Breast.

[16] Merriel SWD, Turner EL, Walsh E, Young GJ, Metcalfe C, Hounsome L (2017). Cross-sectional study evaluating data quality of the National Cancer Registration and Analysis Service (NCRAS) prostate cancer registry data using the Cluster randomised trial of PSA testing for Prostate cancer (CAP). BMJ Open.

[17] Townsend P, Phillimore P, Beattie A. Health and deprivation: inequality and the North. London: Routledge; 1988. 10.4324/9781003368885.

[18] Piernas C, Perez-Cornago A, Gao M, Young H, Pollard Z, Mulligan A (2021). Describing a new food group classification system for UK biobank: analysis of food groups and sources of macro- and micronutrients in 208,200 participants. Eur J Nutr.

[19] Jacobs I, Taljaard-Krugell C, Wicks M, Cubasch H, Joffe M, Laubscher R (2022). Adherence to cancer prevention recommendations is associated with a lower breast cancer risk in black urban South African women. Br J Nutr.

[20] Arthur RS, Wang T, Xue X, Kamensky V, Rohan TE (2020). Genetic Factors, Adherence to Healthy Lifestyle Behavior, and Risk of Invasive Breast Cancer Among Women in the UK Biobank. J Natl Cancer Inst.

[21] Karavasiloglou N, Pestoni G, Kuhn T, Rohrmann S. Adherence to cancer prevention recommendations and risk of breast cancer in situ in the United Kingdom Biobank. Int J Cancer. 2022;151(10):1674–83. 10.1002/ijc.34183 PMC9796371 35723078

[22] Petimar J, Smith-Warner SA, Rosner B, Chan AT, Giovannucci EL, Tabung FK (2019). Adherence to the World Cancer Research Fund/American Institute for Cancer Research 2018 Recommendations for Cancer Prevention and Risk of Colorectal Cancer. Cancer Epidemiol Biomarkers Prev.

[23] Onyeaghala G, Lintelmann AK, Joshu CE, Lutsey PL, Folsom AR, Robien K (2020). Adherence to the World Cancer Research Fund/American Institute for Cancer Research cancer prevention guidelines and colorectal cancer incidence among African Americans and whites: The Atherosclerosis Risk in Communities study. Cancer.

[24] El Kinany K, Huybrechts I, Kampman E, Boudouaya HA, Hatime Z, Mint Sidi Deoula M (2019). Concordance with the World Cancer Research Fund/American Institute for Cancer Research recommendations for cancer prevention and colorectal cancer risk in Morocco: A large, population-based case-control study. Int J Cancer.

[25] Romaguera D, Vergnaud AC, Peeters PH, van Gils CH, Chan DS, Ferrari P (2012). Is concordance with World Cancer Research Fund/American Institute for Cancer Research guidelines for cancer prevention related to subsequent risk of cancer? Results from the EPIC study. Am J Clin Nutr.

[26] Alvarez A, Benjaminsen Borch K, Rylander C (2021). Reproductive factors, use of exogenous hormones, and pancreatic cancer incidence: the Norwegian women and cancer study. Clin Epidemiol.

[27] Teng Y, Saito E, Abe SK, Sawada N, Iwasaki M, Yamaji T (2017). Female reproductive factors, exogenous hormone use, and pancreatic cancer risk: the Japan Public Health Center-based prospective study. Eur J Cancer Prev.

[28] Bravi F, Polesel J, Garavello W, Serraino D, Negri E, Franchin G (2017). Adherence to the World Cancer Research Fund/American Institute for Cancer Research recommendations and head and neck cancers risk. Oral Oncol.

[29] Shams-White MM, Romaguera D, Mitrou P, Reedy J, Bender A, Brockton NT (2020). Further Guidance in Implementing the Standardized 2018 World Cancer Research Fund/American Institute for Cancer Research (WCRF/AICR) Score. Cancer Epidemiol Biomarkers Prev.

[30] Fry A, Littlejohns TJ, Sudlow C, Doherty N, Adamska L, Sprosen T (2017). Comparison of sociodemographic and health-related characteristics of UK Biobank participants with those of the general population. Am J Epidemiol.

[31] Malcomson FC, Parra-Soto S, Lu L, Ho FK, Celis-Morales C, Sharp L, et al. Socio-demographic variation in adherence to the World Cancer Research Fund (WCRF)/American Institute for Cancer Research (AICR) Cancer Prevention Recommendations within the UK Biobank prospective cohort study. J Public Health (Oxf.). In press. 10.1093/pubmed/fdad218 PMC10901269 37986550

[32] van Zutphen M, Boshuizen HC, Kenkhuis MF, Wesselink E, Geijsen A, de Wilt JHW (2021). Lifestyle after colorectal cancer diagnosis in relation to recurrence and all-cause mortality. Am J Clin Nutr.

[33] von Wagner C, Baio G, Raine R, Snowball J, Morris S, Atkin W (2011). Inequalities in participation in an organized national colorectal cancer screening programme: results from the first 2.6 million invitations in England. Int J Epidemiol..

[34] Streiner DL (2015). Best (but oft-forgotten) practices: the multiple problems of multiplicity-whether and how to correct for many statistical tests. Am J Clin Nutr.

[35] Greenwood DC, Hardie LJ, Frost GS, Alwan NA, Bradbury KE, Carter M (2019). Validation of the Oxford WebQ online 24-hour dietary questionnaire using biomarkers. Am J Epidemiol.

[36] Pearce M, Strain T, Kim Y, Sharp SJ, Westgate K, Wijndaele K (2020). Estimating physical activity from self-reported behaviours in large-scale population studies using network harmonisation: findings from UK Biobank and associations with disease outcomes. Int J Behav Nutr Phys Act.

[37] Carter JL, Lewington S, Piernas C, Bradbury K, Key TJ, Jebb SA (2019). Reproducibility of dietary intakes of macronutrients, specific food groups, and dietary patterns in 211 050 adults in the UK Biobank study. J Nutr Sci.

[38] Bradbury KE, Young HJ, Guo W, Key TJ (2018). Dietary assessment in UK Biobank: an evaluation of the performance of the touchscreen dietary questionnaire. J Nutr Sci.

[39] Perez-Cornago A, Pollard Z, Young H, van Uden M, Andrews C, Piernas C (2021). Description of the updated nutrition calculation of the Oxford WebQ questionnaire and comparison with the previous version among 207,144 participants in UK Biobank. Eur J Nutr.

[40] Galante J, Adamska L, Young A, Young H, Littlejohns TJ, Gallacher J (2016). The acceptability of repeat Internet-based hybrid diet assessment of previous 24-h dietary intake: administration of the Oxford WebQ in UK Biobank. Br J Nutr.

