# Supplementary material for: Adherence to the 2018 World Cancer Research Fund (WCRF)/American Institute for Cancer Research (AICR) Cancer Prevention Recommendations and risk of 14 lifestyle-related cancers in the UK Biobank prospective cohort study
Source: BMC Med. 2023 Nov 28;21:407. doi: 10.1186/s12916-023-03107-y (PMC10683228; doi:10.1186/s12916-023-03107-y)
Supplement: Supplementary file 1 — Additional file 1: Supplementary Methods. Calculation of alternative scores for sensitivity analyses. Figure S1. Flow chart of UK Biobank Study participants included in the present study. Table S1. Operationalisation of ‘2018 WCRF/AICR Score’ to assess adherence to the 2018 WCRF/AICR Cancer Prevention Recommendations, as devised by Shams-White et al. (2019). Table S2. Sociodemographic characteristics of UK Biobank participants with a total adherence score, who are included in the present analysis, and those without a total score, who were excluded. Table S3. Total score, lower and upper limits, and corresponding hazard ratios (HR) associated with lowest observed cancer risk for those cancers for which a significant inverse association with total score was observed. Table S4. Associations between total adherence score and risk of all cancers combined and of cancer at individual anatomical sites, stratified according to sex. Table S5. Associations between total adherence score and risk of all cancers combined and of cancer at individual anatomical sites, stratified according to smoking status. Table S6. Associations between total adherence score and risk of all cancers combined and of cancer at individual anatomical sites, adjusting for additional confounders. Table S7. Sensitivity analysis for associations between 1-point increment in total score including fruit juices for the sugar-sweetened drinks score component and risk of all cancers and of cancer at individual anatomical sites. Table S8. Sensitivity analysis for associations between total score including fruit juices for the sugar-sweetened drinks score component, categorised according to score tertiles, and risk of all cancers and of cancers at individual anatomical sites. Table S9. Sensitivity analysis for associations between 1-point increment in total score using original cut-points (based on US guidelines) for alcohol score component and risk of all cancers and of cancer at individual anatomical sites. Tab [file 12916_2023_3107_MOESM1_ESM.docx]

**Additional File 1**

**Supplementary Methods: *Calculation of alternative scores for sensitivity analyses:***

We performed two sensitivity analyses. For the first, we derived a score which included pure fruit juices within the sugar-sweetened beverages score component. Responses to the question “How much of the following did you drink yesterday?’ were used. In addition to the already included sugar-sweetened drinks, the following drinks were used to assess adherence to the recommendation: orange juice (data-field 100190), grapefruit juice (data-field 100200), other pure fruit/vegetable juice (data-field 100210).

For the second analysis, we calculated a score using the cut-offs described in the 2018 WCRF/AICR Score, based on US guidelines (28g of ethanol (2 drinks) and 14 g of ethanol (1 drink) per day for males and females, respectively), to assess adherence to the recommendation to limit alcohol consumption. Units of alcohol consumed per week were multiplied by 8g of ethanol (contained in one unit of alcohol in the UK) and then divided by 14g (amount of ethanol in one standard US drink). Alcohol intake in standard drinks per day was then calculated by dividing this value by 7. The cut-offs were operationalised to allocated points for males and females separately, as detailed in Supplementary Table 1.

**Figure S1: Flow chart of UK Biobank Study participants included in the present study.**

**
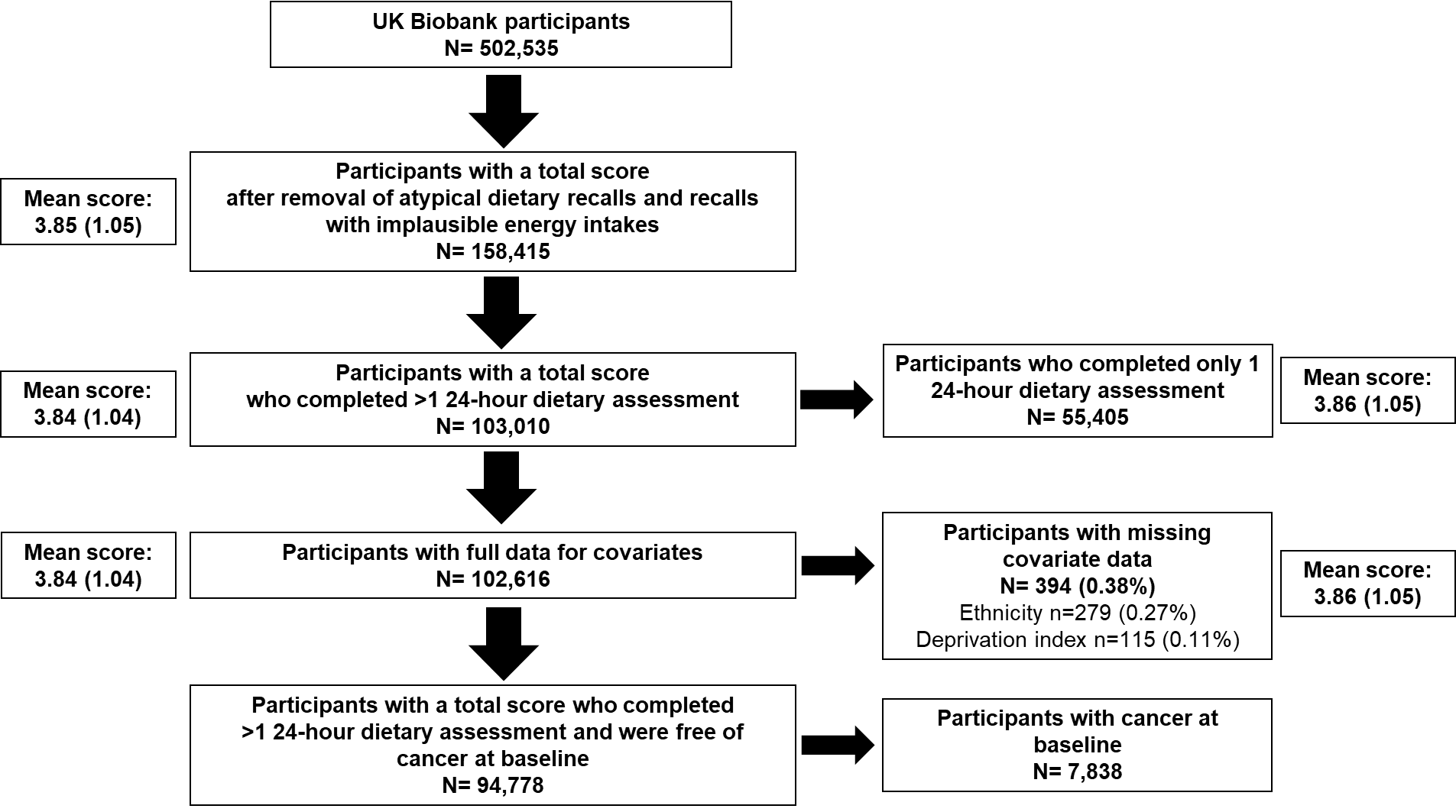
**

**Table S1: Operationalisation of ‘2018 WCRF/AICR Score’ to assess adherence to the 2018 WCRF/AICR Cancer Prevention Recommendations, as devised by Shams-White et al. (2019)(Shams-White et al., 2019)**

| **2018 WCRF/AICR Recommendation** | **Operationalization of Recommendations** | **Points** |
| --- | --- | --- |
| 1. Be a healthy weight | **BMI (kg/m^2^)** |  |
|  | 18.5–24.9 | 0.5 |
|  | 25–29.9 | 0.25 |
|  | <18.5 or ≥30 | 0 |
|  | **Waist circumference (cm (in))** |  |
|  | Men: <94 (<37) Women: <80 (<31.5) | 0.5 |
|  | Men: 94–<102 (37–<40) Women: 80–<88 (31.5–<35) | 0.25 |
|  | Men: ≥102 (≥40) Women: ≥88 (≥35) | 0 |
| 1. Be physically active | **Total moderate-vigorous physical activity (MET min/wk)** |  |
|  | ≥600 | 1 |
|  | 300–<600 | 0.5 |
|  | <300 | 0 |
| 1. Eat a diet rich in wholegrains, vegetables, fruit and beans | **Fruits and vegetables (g/day)** |  |
|  | ≥400 | 0.5 |
|  | 200–<400 | 0.25 |
|  | <200 | 0 |
|  | **Total fibre (g/day) (AOAC definition)** |  |
|  | ≥30 | 0.5 |
|  | 15–<30 | 0.25 |
|  | <15 | 0 |
| 1. Limit consumption of “fast foods” and other processed foods high in fat, starches or sugars | **Percent of total kcal from ultra-processed foods (aUPFs)** |  |
|  | Tertile 1 (lowest) | 1 |
|  | Tertile 2 | 0.5 |
|  | Tertile 3 (highest) | 0 |
| 1. Limit consumption of red and processed meat | **Total red meat and processed meat (g/wk)** |  |
|  | Red meat ≤500 and processed meat <21 | 1 |
|  | Red meat ≤500 and processed meat 21–<100 | 0.5 |
|  | Red meat >500 or processed meat ≥100 | 0 |
| 1. Limit consumption of sugar-sweetened drinks | **Total sugar-sweetened drinks (g/day):** |  |
|  | 0 | 1 |
|  | >0–≤250 | 0.5 |
|  | >250 | 0 |
| 1. Limit alcohol consumption | **Total ethanol (UK guidelines) (units/week)):** |  |
|  | 0 | 1 |
|  | ≤14 | 0.5 |
|  | >14 | 0 |
|  | **Sensitivity analysis (US guidelines) (ethanol, g/day)** |  |
|  | 0 | 1 |
|  | Men: >0–≤28 (2 drinks) and women: ≤14 (1 drink) | 0.5 |
|  | Men: >28 (2 drinks) and women: >14 (1 drink) | 0 |

**Table S2:** **Sociodemographic characteristics of UK Biobank participants with a total adherence score, who are included in the present analysis, and those without a total score, who were excluded**

|  | **Participants with a total score included in the present analysis** | **Participants without a total score excluded from this analysis** |
| --- | --- | --- |
| n | 94,778 | 407,694 |
| Females (%) | 50,628 (53.4) | 222,763 (54.6) |
| Age (years) | 55.8 (7.9) | 56.6 (8.1) |
| Townsend deprivation index | -1.67 (2.82) | -1.21 (3.15) |
| Ethnicity (%) |  |  |
| White | 91,729 (96.8) | 380,934 (94.1) |
| Mixed | 1,072 (1.1) | 6,444 (1.6) |
| South Asian | 968 (1.0) | 8,914 (2.2) |
| Black | 761 (0.8) | 7,300 (1.8) |
| Chinese | 248 (0.3) | 1,326 (0.3) |
| Smoking (%) |  |  |
| Never | 54,978 (58.0) | 218,534 (53.6) |
| Former | 33,269 (35.1) | 139,781 (34.3) |
| Current | 6,389 (6.7) | 46,588 (11.4) |
| Education (%) |  |  |
| College or University degree | 45,806 (48.3) | 115,370 (28.6) |
| A levels/AS levels or equivalent | 12,826 (13.53 | 42,498 (10.5) |
| O levels/GCSEs or equivalent | 18,137 (19.1) | 87,063 (21.6) |
| CSEs or equivalent | 3,127 (3.3) | 23,760 (5.9) |
| NVQ or HND or HNC or equivalent | 4,681 (4.9) | 28,049 (7.0) |
| Other professional qualifications | 4,486 (4.7) | 21,319 (5.3) |
| None of the above | 5,527 (5.8) | 79,748 (19.8) |
| Do not know/prefer not to answer | 188 (0.2) | 5,305 (1.3) |

Data are presented as means and standard deviation (SD) or ‘n’ and percentage (%).

Data missing for subset of participants with a total score: 0.2% smoking status. Data missing for participants without score: 0.02% age, 0.2% Townsend Deprivation Index, 0.7% ethnicity, 0.7% smoking status, 1.1% education.

A levels: Advanced levels, AS levels: Advance Subsidiary levels, CSE: Certificate of Secondary Education, GCSE: General Certificate of Secondary Education, HNC: Higher National Certificate, HND: Higher National Diploma, NVQ: National Vocational Qualifications, O levels: General Certificate of Education Ordinary Level

**Table S3: Total score, lower and upper limits, and corresponding hazard ratios (HR) associated with lowest observed cancer risk for those cancers for which a significant inverse association with total score was observed**

|  | **Score with lowest observed cancer risk** | | **Lower limit** | | **Upper limit** | |
| --- | --- | --- | --- | --- | --- | --- |
| **Cancer** | **Score (points)** | **Corresponding HR** | **Score (points)** | **Corresponding HR** | **Score (points)** | **Corresponding HR** |
| Overall | 7.00 | 0.68 | 5.75 | 0.73 | 7.00 | 0.68 |
| Breast | 7.00 | 0.54 | 5.75 | 0.60 | 7.00 | 0.54 |
| Colorectal | 5.00 | 0.84 | 1.5 | 1.09 | 5.00 | 0.84 |
| Kidney | 7.00 | 0.48 | 5.00 | 0.62 | 7.00 | 0.48 |
| Oesophageal | 5.25 | 0.39 | 4.25 | 0.46 | 5.25 | 0.39 |
| Ovarian | 7.00 | 0.23 | 4.5 | 0.27 | 7.00 | 0.23 |
| Liver | 7.00 | 0.19 | 5.00 | 0.29 | 7.00 | 0.19 |
| Gallbladder | 7.00 | 0.13 | 5.4 | 0.20 | 7.00 | 0.13 |

The lower and upper limits correspond to the range of scores with 95% CIs compatible with the score with lowest observed cancer risk.

**Table S4 Associations between total adherence score and risk of all cancers combined and of cancer at individual anatomical sites, stratified according to sex**

|  |  | **Females** | | | | | | |  | **Males** | | | | | |
| --- | --- | --- | --- | --- | --- | --- | --- | --- | --- | --- | --- | --- | --- | --- | --- |
|  |  | **Continuous** |  | **Mid Score**  **(3.75 – 4.25)** | | **Higher score**  **(4.5 – 7)** | |  | | **Continuous** | | **Mid Score**  **(3.75 – 4.25)** | | **Higher score**  **(4.5 – 7)** | |
| **Cancer site** | **Cases (n)** | **Model 2 HR**  **(95% CI)** | **P-value** | **Model 2 HR**  **(95% CI)** | **P-value** | **Model 2 HR**  **(95% CI)** | **P-value** | **Cases (n)** | | **Model 2 HR**  **(95% CI)** | **P-value** | **Model 2 HR**  **(95% CI)** | **P-value** | **Model 2 HR**  **(95% CI)** | **P-value** |
| All cancers combined | 3,303 | **0.91 (0.88; 0.94)** | **<0.001** | **0.88 (0.81; 0.96)** | **0.004** | **0.82 (0.76; 0.89)** | **<0.001** | 3,985 | | **0.94 (0.91; 0.97)** | **<0.001** | 0.95 (0.89; 1.03) | 0.200 | **0.85 (0.78; 0.92)** | **<0.001** |
| Prostate |  |  |  |  |  |  |  | 1,812 | | 1.00 (0.96; 1.05) | 0.959 | 1.05 (0.95; 1.18) | 0.306 | 0.96 (0.85; 1.08) | 0.499 |
| Breast | 1,438 | **0.90 (0.86; 0.95)** | **<0.001** | **0.82 (0.72; 0.94)** | **0.003** | **0.82 (0.73; 0.93)** | **0.002** |  | |  |  |  |  |  |  |
| Colorectal | 332 | 0.92 (0.82; 1.02) | 0.114 | 0.76 (0.58; 1.01) | 0.058 | 0.86 (0.67; 1.10) | 0.221 | 530 | | **0.90 (0.82; 0.98)** | **0.014** | 0.83 (0.67; 1.02) | 0.070 | **0.72 (0.57; 0.91)** | **0.007** |
| Lung | 204 | 1.00 (0.87; 1.15) | 0.973 | 1.12 (0.80; 1.57) | 0.524 | 0.85 (0.60; 1.18) | 0.328 | 226 | | **0.87 (0.76; 0.99)** | **0.034** | 0.94 (0.69; 1.28) | 0.706 | 0.77 (0.53; 1.12) | 0.171 |
| Bladder | 20 | 0.99 (0.64; 1.53) | 0.954 | 1.97 (0.66; 5.90) | 0.874 | 0.91 (0.28; 2.99) | 0.874 | 121 | | 0.89 (0.74; 1.07) | 0.224 | **0.61 (0.38; 0.97)** | **0.037** | 0.74 (0.46; 1.19) | 0.215 |
| Kidney | 60 | **0.77 (0.60; 0.99)** | **0.044** | **0.93 (0.52; 1.68)** | **0.820** | **0.48 (0.25; 0.92)** | **0.027** | 161 | | **0.84 (0.72; 0.98)** | **0.029** | 0.80 (0.55; 1.17) | 0.257 | 0.75 (0.49; 1.13) | 0.170 |
| Pancreas | 95 | 0.93 (0.76; 1.15) | 0.530 | 0.90 (0.54; 1.50) | 0.690 | 0.80 (0.50; 1.30) | 0.366 | 118 | | **0.83 (0.69; 0.99)** | **0.042** | **0.62 (0.39; 0.99)** | **0.045** | 0.72 (0.45; 1.16) | 0.181 |
| Uterus | 212 | 0.92 (0.81; 1.05) | 0.227 | 0.99 (0.71; 1.39) | 0.964 | 0.83 (0.60; 1.14) | 0.250 |  | |  |  |  |  |  |  |
| Oesophagus | 43 | 1.09 (0.80; 1.48) | 0.581 | 1.51 (0.69; 3.29) | 0.299 | 1.14 (0.53; 2.46) | 0.738 | 108 | | **0.76 (0.62; 0.92)** | **0.004** | 0.78 (0.50; 1.22) | 0.270 | **0.48 (0.27; 0.88)** | **0.016** |
| Head and Neck | 52 | 1.20 (0.91; 1.58) | 0.203 | 1.26 (0.59; 2.68) | 0.550 | 1.62 (0.83; 3.17) | 0.159 | 77 | | 1.22 (0.98; 1.53) | 0.079 | 1.67 (1.00; 2.79) | 0.051 | 1.37 (0.77; 2.45) | 0.286 |
| Ovary | 147 | **0.76 (0.65; 0.90)** | **0.001** | 0.69 (0.46; 1.03) | 0.070 | **0.57 (0.39; 0.84)** | **0.005** |  | |  |  |  |  |  |  |
| Stomach | 30 | 1.07 (0.75; 1.54) | 0.698 | 1.36 (0.56; 3.28) | 0.499 | 0.88 (0.36; 2.17) | 0.780 | 69 | | 0.94 (0.74; 1.19) | 0.610 | 0.90 (0.50; 1.60) | 0.708 | 1.07 (0.59; 1.93) | 0.828 |
| Liver | 32 | 0.92 (0.65; 1.30) | 0.652 | 0.91 (0.38; 2.20) | 0.837 | 0.86 (0.38; 1.96) | 0.727 | 50 | | **0.70 (0.53; 0.93)** | **0.012** | 0.75 (0.39; 1.47) | 0.407 | 0.55 (0.24; 1.24) | 0.150 |
| Gallbladder | 21 | 0.86 (0.56; 1.32) | 0.483 | 1.21 (0.41; 3.61) | 0.731 | 0.97 (0.34; 2.81) | 0.959 | 28 | | **0.61 (0.42; 0.88)** | **0.008** | 0.45 (0.17; 1.21) | 0.113 | 0.31 (0.09; 1.05) | 0.060 |

Data are presented as hazard ratio (HR) with 95% confidence intervals in parentheses (95% CIs) per 1-point increment in score for continuous analysis, and for mid (3.75 – 4.25) and higher (4.5 – 7 points) score tertiles vs. the lowest score tertile (0 – 3.5 points) as the reference group for categorical analysis. Data represent those derived from Model 2 (minus sex), adjusted for age, ethnicity, Townsend deprivation index, and smoking status.

**Table S5 Associations between total adherence score and risk of all cancers combined and of cancer at individual anatomical sites, stratified according to smoking status**

|  |  | **Continuous** |  | **Mid Score**  **(3.75 – 4.25)** | | **Higher score**  **(4.5 – 7)** | | |
| --- | --- | --- | --- | --- | --- | --- | --- | --- |
| **Cancer site** | **Cases (n)** | **HR**  **(95% CI)** | **P-value** | **HR**  **(95% CI)** | **P-value** | **HR**  **(95% CI)** | **P-value** |  |
| *Never smokers* | | | | | | | |  |
| All cancers combined | 3,762 | **0.92 (0.90; 0.95)** | **<0.001** | **0.91 (0.84; 0.98)** | **0.016** | **0.83 (0.77; 0.90)** | **<0.001** |  |
| Prostate | 953 | 0.99 (0.93; 1.06) | 0.841 | 1.13 (0.97; 1.31) | 0.108 | 0.93 (0.79; 1.09) | 0.373 |  |
| Breast | 865 | **0.92 (0.86; 0.98)** | **0.012** | **0.84 (0.71; 0.99)** | **0.040** | **0.82 (0.70; 0.96)** | **0.012** |  |
| Colorectal | 400 | **0.86 (0.78; 0.95)** | **0.003** | **0.75 (0.59; 0.95)** | **0.019** | **0.67 (0.52; 0.85)** | **0.001** |  |
| Lung | 80 | 0.92 (0.74; 1.15) | 0.462 | 0.90 (0.52; 1.56) | 0.714 | 0.87 (0.52; 1.48) | 0.616 |  |
| Bladder | 48 | 0.85 (0.64; 1.12) | 0.253 | 0.63 (0.30; 1.32) | 0.218 | 0.70 (0.36; 1.40) | 0.316 |  |
| Kidney | 109 | **0.72 (0.60; 0.87)** | **0.001** | 0.75 (0.48; 1.17) | 0.202 | **0.44 (0.26; 0.74)** | **0.002** |  |
| Pancreas | 107 | 0.88 (0.72; 1.06) | 0.172 | 0.72 (0.45; 1.17) | 0.187 | 0.77 (0.49; 1.21) | 0.264 |  |
| Uterus | 139 | 0.87 (0.74; 1.03) | 0.112 | 0.92 (0.60; 1.40) | 0.699 | 0.83 (0.56; 1.23) | 0.352 |  |
| Oesophagus | 56 | 0.98 (0.75; 1.27) | 0.857 | 1.21 (0.64; 2.28) | 0.555 | 0.94 (0.48; 1.82) | 0.852 |  |
| Head and Neck | 50 | 1.18 (0.89; 1.56) | 0.242 | 1.41 (0.69; 2.90) | 0.348 | 1.63 (0.82; 3.23) | 0.161 |  |
| Ovary | 87 | **0.69 (0.56; 0.84)** | **<0.001** | **0.55 (0.32; 0.94)** | **0.029** | **0.51 (0.31; 0.84)** | **0.008** |  |
| Stomach | 46 | 0.82 (0.62; 1.10) | 0.190 | 0.95 (0.48; 1.87) | 0.880 | 0.70 (0.33; 1.47) | 0.340 |  |
| Liver | 39 | 0.75 (0.55; 1.03) | 0.072 | 0.46 (0.12; 1.08) | 0.075 | 0.63 (0.30; 1.32) | 0.222 |  |
| Gallbladder | 29 | 0.69 (0.48; 0.99) | 0.046 | 0.52 (0.20; 1.35) | 0.181 | 0.57 (0.24; 1.36) | 0.204 |  |
| *Former smokers* | | | | | | | |  |
| All cancers combined | 2,909 | **0.92 (0.89; 0.96)** | **<0.001** | 0.95 (0.87; 1.04) | 0.244 | **0.82 (0.75; 0.90)** | **<0.001** |  |
| Prostate | 748 | 0.99 (0.92; 1.07) | 0.858 | 0.93 (0.78; 1.10) | 0.406 | 0.96 (0.80; 1.16) | 0.688 |  |
| Breast | 473 | **0.88 (0.80; 0.96** | **0.004** | 0.80 (0.64; 1.01) | 0.066 | 0.84 (0.68; 1.03) | 0.096 |  |
| Colorectal | 387 | 0.92 (0.83; 1.02) | 0.104 | 0.81 (0.63; 1.04) | 0.235 | 0.86 (0.67; 1.10) | 0.235 |  |
| Lung | 225 | 0.90 (0.79; 1.03) | 0.134 | 1.04 (0.47; 0.94) | 0.820 | **0.66 (0.47; 0.94)** | **0.020** |  |
| Bladder | 78 | 0.94 (0.75; 1.19) | 0.626 | 0.85 (0.50; 1.45) | 0.550 | 0.69 (0.37; 1.29) | 0.247 |  |
| Kidney | 92 | 0.91 (0.74; 1.12) | 0.387 | 0.89 (0.54; 1.47) | 0.653 | 0.94 (0.56; 1.57) | 0.801 |  |
| Pancreas | 80 | **0.80 (0.64; 0.99)** | **0.046** | 0.70 (0.41; 1.20) | 0.190 | 0.58 (0.33; 1.05) | 0.071 |  |
| Uterus | 67 | 1.09 (0.86; 1.39) | 0.481 | 1.34 (0.73; 2.43) | 0.342 | 0.99 (0.55; 1.80) | 0.980 |  |
| Oesophagus | 73 | 0.82 (0.65; 1.04) | 0.105 | 0.77 (0.44; 1.34) | 0.349 | 0.60 (0.32; 1.14) | 0.121 |  |
| Head and Neck | 63 | 1.24 (0.96; 1.59) | 0.094 | 1.65 (0.92; 2.98) | 0.365 | 1.34 (0.71; 2.52) | 0.365 |  |
| Ovary | 54 | 0.88 (0.67; 1.15) | 0.341 | 0.82 (0.42; 1.60) | 0.559 | 0.66 (0.35; 1.26) | 0.207 |  |
| Stomach | 44 | 1.19 (0.88; 1.61) | 0.264 | 1.47 (0.72; 3.00) | 0.285 | 1.53 (0.73; 3.20) | 0.259 |  |
| Liver | 34 | 0.77(0.55; 1.08) | 0.125 | 0.97 (0.45; 2.09) | 0.939 | 0.53 (0.19; 1.44) | 0.211 |  |
| Gallbladder | 18 | 0.76 (0.48; 1.21) | 0.244 | 1.10 (0.39; 3.13) | 0.854 | 0.54 (0.14; 2.07) | 0.372 |  |
| *Current smokers* | | | | | | | |  |
| All cancers combined | 617 | 0.95 (0.87; 1.03) | 0.177 | 0.87 (0.72; 1.06) | 0.170 | 0.99 (0.81; 1.21) | 0.921 |  |
| Prostate | 111 | 1.11 (0.92; 1.34) | 0.294 | 1.36 (0.88; 2.09) | 0.167 | 1.34 (0.81; 2.23) | 0.259 |  |
| Breast | 100 | 0.87 (0.72; 1.05) | 0.138 | 0.75 (0.46; 1.23) | 0.263 | 0.82 (0.52; 1.30) | 0.400 |  |
| Colorectal | 75 | 1.06 (0.84; 1.33) | 0.631 | 0.92 (0.52; 1.64) | 0.785 | 1.28 (0.73; 2.25) | 0.396 |  |
| Lung | 125 | 0.97 (0.81; 1.16) | 0.759 | 1.06 (0.69; 1.62) | 0.784 | 1.03 (0.65; 1.63) | 0.891 |  |
| Bladder | 15 | 0.91 (0.55; 1.52) | 0.731 | 0.46 (0.10; 2.13) | 0.324 | 0.98 (0.26; 3.62) | 0.973 |  |
| Kidney | 20 | 1.02 (0.66; 1.58) | 0.942 | 1.25 (0.46; 3.38) | 0.667 | 0.81 (0.22; 2.94) | 0.745 |  |
| Pancreas | 26 | 1.14 (0.78; 1.68) | 0.504 | 0.91 (0.34; 2.40) | 0.846 | 1.22 (0.47; 3.12) | 0.686 |  |
| Uterus | 6 | 0.48 (0.23; 1.03) | 0.060 | 0.29 (0.03; 2.50) | 0.261 | NA | NA |  |
| Oesophagus | 22 | **0.60 (0.39; 0.93)** | **0.024** | 0.87 (0.34; 2.24) | 0.779 | NA | NA |  |
| Head and Neck | 16 | 1.17 (0.72; 1.91) | 0.519 | 1.21 (0.35; 4.16) | 0.762 | 1.78 (0.55; 5.76) | 338 |  |
| Ovary | 6 | 0.82 (0.38; 1.76) | 0.604 | 2.13 (0.36; 12.8) | 0.407 | 0.64 (0.06; 7.10) | 0.718 |  |
| Stomach | 9 | 0.96 (0.49; 1.88) | 0.911 | NA | NA | 0.82 (0.16; 4.14) | 0.812 |  |
| Liver | 9 | 0.99 (0.52; 1.89) | 0.969 | 2.88 (0.64; 13.0) | 0.168 | 1.77 (0.29; 10.9) | 0.537 |  |
| Gallbladder | 2 | 0.40 (0.09; 1.91) | 0.252 | NA | NA | NA | NA |  |

Data are presented as hazard ratio (HR) with 95% confidence intervals in parentheses (95% CIs) per 1-point increment in score for continuous analysis, and for mid (3.75 – 4.25) and higher (4.5 – 7 points) score tertiles vs. the lowest score tertile (0 – 3.5 points) as the reference group for categorical analysis. Data represent those derived from Model 2 (minus sex), adjusted for age, ethnicity, Townsend deprivation index, and smoking status. ^1^ NA: insufficient ‘n’ for analysis.

**Table S6: Associations between total adherence score and risk of all cancers combined and of cancer at individual anatomical sites, adjusting for additional confounders**

|  | **Continuous** |  | **Low score**  **(0-3.25)** | **Mid Score**  **(3.75 – 4.25)** | | **Higher score**  **(4.5 – 7)** | |
| --- | --- | --- | --- | --- | --- | --- | --- |
| **Cancer site** | **HR**  **(95% CI)** | **P-value** | **HR (95% CI)** | **HR**  **(95% CI)** | **P-value** | **HR**  **(95% CI)** | **P-value** |
| All cancers combined | **0.93 (0.91; 0.95)** | **<0.001** | 1.00 (ref) | **0.92 (0.87; 0.98)** | **0.006** | **0.84 (0.79; 0.89)** | **<0.001** |
| Prostate | 0.99 (0.95; 1.04) | 0.729 | 1.00 (ref) | 1.04 (0.93; 1.16) | 0.469 | 0.94 (0.84; 1.06) | 0.335 |
| Breast | **0.81 (0.72; 0.91)** | **<0.001** | 1.00 (ref) | **0.66 (0.49; 0.89)** | **0.006** | **0.72 (0.55; 0.94)** | **0.016** |
| Colorectal | **0.87 (0.82; 0.93)** | **<0.001** | 1.00 (ref) | **0.76 (0.65; 0.90)** | **0.001** | **0.73 (0.62; 0.86)** | **<0.001** |
| Lung | 0.96 (0.87; 1.06) | 0.392 | 1.00 (ref) | 1.07 (0.85; 1.34) | 0.565 | 0.85 (0.66; 1.09) | 0.204 |
| Bladder | 0.91 (0.77; 1.08) | 0.296 | 1.00 (ref) | 0.74 (0.49; 1.12) | 0.160 | 0.73 (0.47; 1.13) | 0.160 |
| Kidney | **0.85 (0.74; 0.97)** | **0.018** | 1.00 (ref) | 0.90 (0.66; 1.24) | 0.525 | **0.68 (0.47; 0.97)** | **0.034** |
| Pancreas | 0.89 (0.78; 1.02) | 0.101 | 1.00 (ref) | 0.76 (0.54; 1.07) | 0.113 | 0.78 (0.55; 1.09) | 0.144 |
| Uterus | 0.90 (0.74; 1.10) | 0.305 | 1.00 (ref) | 0.93 (0.58; 1.48) | 0.752 | 0.76 (0.48; 1.19) | 0.229 |
| Oesophagus | 0.87 (0.74; 1.02) | 0.095 | 1.00 (ref) | 0.96 (0.65; 1.40) | 0.825 | 0.70 (0.45; 1.09) | 0.118 |
| Head and Neck | **1.22 (1.02; 1.46)** | **0.027** | 1.00 (ref) | 1.52 (0.99; 2.33) | 0.056 | 1.54 (1.00; 2.38) | 0.052 |
| Ovary | 0.91 (0.73; 1.15) | 0.438 | 1.00 (ref) | 0.86 (0.49; 1.50) | 0.588 | 0.78 (0.45; 1.33) | 0.362 |
| Stomach | 0.97 (0.79; 1.19) | 0.774 | 1.00 (ref) | 0.98 (0.61; 1.60) | 0.951 | 0.96 (0.58; 1.58) | 0.872 |
| Liver | 0.82 (0.66; 1.02) | 0.075 | 1.00 (ref) | 0.86 (0.51; 1.46) | 0.579 | 0.75 (0.43; 1.32) | 0.320 |
| Gallbladder | **0.70 (0.53; 0.94)** | **0.016** | 1.00 (ref) | 0.70 (0.45; 1.40) | 0.310 | 0.56 (0.27; 1.17) | 0.125 |

Data are presented as hazard ratio (HR) with 95% confidence intervals in parentheses (95% CIs) per 1-point increment in score for continuous analysis, and for mid (3.75 – 4.25) and higher (4.5 – 7 points) score tertiles vs. the lowest score tertile (0 – 3.5 points) as the reference group for categorical analysis.

Data represent those adjusted for age, sex, ethnicity, Townsend deprivation index, smoking status and, additionally, mean total daily energy intake, multimorbidity, education, number of 24-hour dietary assessments completed. For prostate, breast, lung, and colorectal cancers, statistical models were additionally adjusted for family history of that cancer. For female cancers (breast, uterine, and ovarian), statistical models were additionally adjusted for menopausal status, use of oral contraceptives, use of hormone replacement therapy, age of menarche, age at first birth, and parity.

**Table S7: Sensitivity analysis for associations between 1-point increment in total score including fruit juices for the sugar-sweetened drinks score component and risk of all cancers and of cancer at individual anatomical sites**

| **Cancer site** | **Model 1**  **HR (95% CI)** | **P value** | **Model 2 HR**  **(95% CI)** | **P-value** |
| --- | --- | --- | --- | --- |
| All cancers combined | **0.92 (0.90; 0.94)** | **<0.001** | **0.92 (0.90; 0.94)** | **<0.001** |
| Prostate | 1.00 (0.96; 1.05) | 0.871 |  |  |
| Breast | **0.89 (0.85; 0.94)** | **<0.001** | **0.90 (0.85; 0.94)** | **<0.001** |
| *Pre-Menopausal* | **0.84 (0.73; 0.98)** | **0.029** | **0.84 (0.72; 0.98)** | **0.027** |
| *Post-Menopausal* | **0.89 (0.85; 0.94)** | **<0.001** | **0.89 (0.85; 0.94)** | **<0.001** |
| Colorectal | **0.88 (0.82; 0.94)** | **<0.001** | **0.89 (0.83; 0.95)** | **<0.001** |
| *Colon* | **0.81 (0.74; 0.88)** | **<0.001** | **0.81 (0.75; 0.88)** | **<0.001** |
| *Distal* | **0.73 (0.65; 0.83)** | **<0.001** | **0.74 (0.65; 0.84)** | **<0.001** |
| *Proximal* | **0.86 (0.77; 0.97)** | **0.010** | **0.87 (0.78; 0.97)** | **0.016** |
| *Rectum* | 1.00 (0.90; 1.11) | 0.993 | 1.02 (0.92; 1.13) | 0.726 |
| Lung | **0.87 (0.79; 0.95)** | **0.003** | 0.93 (0.85; 1.02) | 0.144 |
| *Current smoker* | 1.00 (0.83; 1.19) | 0.978 |  |  |
| *Previous smoker* | 0.93 (0.81; 1.06) | 0.258 |  |  |
| *Never smoked* | 0.84 (0.68; 1.05) | 0.131 |  |  |
| Bladder | 0.87 (0.73; 1.03) | 0.097 | 0.89 (0.75; 1.06) | 0.194 |
| Kidney | **0.85 (0.74; 0.96)** | **0.013** | **0.85 (0.74; 0.97)** | **0.017** |
| Pancreas | 0.88 (0.77; 1.01) | 0.064 | 0.89 (0.78; 1.02) | 0.095 |
| Uterus | 0.93 (0.81; 1.06) | 0.265 | 0.92 (0.81; 1.05) | 0.232 |
| Oesophagus | **0.83 (0.71; 0.98)** | **0.026** | 0.86 (0.73; 1.01) | 0.062 |
| *Current smoker* | 0.67 (0.43; 1.04) | 0.077 |  |  |
| *Previous smoker* | 0.84 (0.66; 1.06) | 0.143 |  |  |
| *Never smoked* | 0.98 (0.75; 1.27) | 0.862 |  |  |
| Head and Neck | **1.21 (1.02; 1.44)** | **0.031** | **1.24 (1.04; 1.48)** | **0.014** |
| *Current smoker* | 1.31 (0.80; 2.14) | 0.287 |  |  |
| *Previous smoker* | 1.23 (0.96; 1.58) | 0.097 |  |  |
| *Never smoked* | 1.22 (0.93; 1.62) | 0.157 |  |  |
| Ovary | **0.76 (0.65; 0.89)** | **0.001** | **0.76 (0.65; 0.89)** | **0.001** |
| Stomach | 0.95 (0.78; 1.16) | 0.625 | 0.96 (0.79; 1.18) | 0.711 |
| Liver | **0.79 (0.63; 0.97)** | **0.028** | **0.79 (0.64; 0.99)** | **0.036** |
| Gallbladder | **0.71 (0.54; 0.94)** | **0.017** |  |  |

Data are presented as hazard ratio (HR) with 95% confidence intervals in parentheses (95% CIs) per 1-point increment in score. Model 1 was adjusted for age, sex, Townsend deprivation index and ethnicity. Model 2 was additionally adjusted for smoking status.

**Table S8: Sensitivity analysis for associations between total score including fruit juices for the sugar-sweetened drinks score component, categorised according to score tertiles, and risk of all cancers and of cancers at individual anatomical sites**

|  |  |  | **Low score (0-3.25)** | **Higher score (4.25 – 7)** | | | |
| --- | --- | --- | --- | --- | --- | --- | --- |
| **Cancer site** | **Total** | **Events** | **HR (95% CI)** | **Model 1 HR (95% CI)** | **P value** | **Model 2 HR (95% CI)** | **P value** |
| All cancers combined | 93,630 | 7,296 | 1.00 (ref) | **0.82 (0.78; 0.87)** | **<0.001** | **0.84 (0.87; 0.97)** | **0.003** |
| Prostate | 43,851 | 1,818 | 1.00 (ref) | 1.00 (0.89; 1.12) | 0.977 |  |  |
| Breast | 50,337 | 1,438 | 1.00 (ref) | **0.86 (0.76; 0.97)** | **0.014** | **0.86 (0.76; 0.97)** | **0.016** |
| *Pre-Menopausal* | 1,108 | 154 | 1.00 (ref) | 0.70 (0.49; 1.11) | 0.147 | 0.69 (0.48; 1.00) | 0.051 |
| *Post-Menopausal* | 49,229 | 1,284 | 1.00 (ref) | **0.86 (0.76; 0.98)** | **0.026** | **0.87 (0.76; 0.99)** | **0.029** |
| Colorectal | 94,656 | 862 | 1.00 (ref) | **0.71 (0.60; 0.84)** | **<0.001** | **0.73 (0.61; 0.86)** | **<0.001** |
| *Colon* | 94,698 | 575 | 1.00 (ref) | **0.61 (0.50; 0.75)** | **<0.001** | **0.62 (0.50; 0.77)** | **<0.001** |
| *Distal* | 94,734 | 242 | 1.00 (ref) | **0.51 (0.37; 0.72)** | **<0.001** | **0.53 (0.38; 0.73)** | **<0.001** |
| *Proximal* | 94,748 | 303 | 1.00 (ref) | **0.68 (0.51; 0.90)** | **0.007** | **0.69 (0.52; 0.92)** | **0.011** |
| *Rectum* | 94,734 | 342 | 1.00 (ref) | 0.92 (0.71; 1.20) | 0.533 | 0.96 (0.74; 1.25) | 0.758 |
| Lung | 94,760 | 431 | 1.00 (ref) | **0.73 (0.57; 0.93)** | **0.012** | 0.87 (0.68; 1.11) | 0.255 |
| *Current smoker* | 6,387 | 125 | 1.00 (ref) | 1.26 (0.79; 2.02) | 0.327 |  |  |
| *Previous smoker* | 33,260 | 225 | 1.00 (ref) | 0.75 (0.53; 1.06) | 0.101 |  |  |
| *Never smoked* | 54,971 | 80 | 1.00 (ref) | 0.71 (0.42; 1.20) | 0.204 |  |  |
| Bladder | 94,756 | 141 | 1.00 (ref) | **0.63 (0.40; 0.99)** | **0.045** | 0.68 (0.43; 1.06) | 0.090 |
| Kidney | 94,754 | 221 | 1.00 (ref) | **0.68 (0.48; 0.95)** | **0.024** | **0.69 (0.49; 0.96)** | **0.029** |
| Pancreas | 94,770 | 213 | 1.00 (ref) | 0.81 (0.59; 1.12) | 0.198 | 0.83 (0.60; 1.15) | 0.264 |
| Uterus | 50,588 | 212 | 1.00 (ref) | 0.83 (0.61; 1.15) | 0.263 | 0.82 (0.60; 1.13) | 0.234 |
| Oesophagus | 94,765 | 151 | 1.00 (ref) | **0.59 (0.38; 0.92)** | **0.021** | **0.63 (0.41; 0.99)** | **0.046** |
| *Current smoker* | 6,388 | 22 | 1.00 (ref) | 0.23 (0.03; 1.74) | 0.155 |  |  |
| *Previous smoker* | 33,260 | 73 | 1.00 (ref) | **0.45 (0.22; 0.92)** | **0.028** |  |  |
| *Never smoked* | 54,975 | 56 | 1.00 (ref) | 1.12 (0.57; 2.19) | 0.737 |  |  |
| Head and Neck | 94,763 | 129 | 1.00 (ref) | **1.54 (1.02; 2.35)** | **0.042** | **1.64 (1.08; 2.50)** | **0.021** |
| *Current smoker* | 6,388 | 16 | 1.00 (ref) | 1.44 (0.45; 4.55) | 0.537 |  |  |
| *Previous smoker* | 33,259 | 63 | 1.00 (ref) | 1.24 (0.67; 2.30) | 0.488 |  |  |
| *Never smoked* | 54,974 | 50 | 1.00 (ref) | **2.43 (1.20; 4.94)** | **0.014** |  |  |
| Ovary | 50,603 | 147 | 1.00 (ref) | **0.59 (0.40; 0.85)** | **0.005** | **0.58 (0.40; 0.85)** | **0.005** |
| Stomach | 94, 770 | 99 | 1.00 (ref) | 1.01 (0.62; 1.63) | 0.975 | 1.04 (0.64; 1.67) | 0.885 |
| Liver | 94,773 | 82 | 1.00 (ref) | **0.55 (0.30; 0.99)** | **0.045** | 0.56 (0.31; 1.01) | 0.056 |
| Gallbladder | 94,777 | 49 | 1.00 (ref) | 0.49 (0.23; 1.03) | 0.059 |  |  |

Data are presented as hazard ratio (HR) with 95% confidence intervals in parentheses (95% CIs). Lowest score tertile was the reference group. Model 1 was adjusted for age, sex, Townsend deprivation index and ethnicity. Model 2 was additionally adjusted for smoking status.

**Table S9: Sensitivity analysis for associations between 1-point increment in total score using original cut-points (based on US guidelines) for alcohol score component and risk of all cancers and of cancer at individual anatomical sites**

| **Cancer site** | **Model 1**  **HR (95% CI)** | **P value** | **Model 2 HR**  **(95% CI)** | **P-value** |
| --- | --- | --- | --- | --- |
| All cancers combined | **0.92 (0.90; 0.94)** | **<0.001** | **0.93 (0.91; 0.95)** | **<0.001** |
| Prostate | 1.02 (0.97; 1.06) | 0.505 |  |  |
| Breast | **0.90 (0.85; 0.94)** | **<0.001** | **0.90 (0.86; 0.95)** | **<0.001** |
| *Pre-Menopausal* | **0.84 (0.73; 0.98)** | **0.024** | **0.84 (0.73; 0.98)** | **0.022** |
| *Post-Menopausal* | **0.90 (0.85; 0.95)** | **<0.001** | **0.90 (0.85; 0.95)** | **<0.001** |
| Colorectal | **0.89 (0.83; 0.95)** | **<0.001** | **0.90 (0.84; 0.96)** | **0.002** |
| *Colon* | **0.81 (0.75; 0.88)** | **<0.001** | **0.82 (0.76; 0.89)** | **<0.001** |
| *Distal* | **0.72 (0.64; 0.82)** | **<0.001** | **0.73 (0.64; 0.83)** | **<0.001** |
| *Proximal* | **0.89 (0.79; 0.99)** | **0.035** | 0.89 (0.80; 1.00) | 0.052 |
| *Rectum* | 1.02 (0.92; 1.14) | 0.699 | 1.04 (0.94; 1.16) | 0.456 |
| Lung | **0.85 (0.78; 0.94)** | **0.001** | 0.92 (0.84; 1.01) | 0.094 |
| *Current smoker* | 0.95 (0.80; 1.13) | 0.567 |  |  |
| *Previous smoker* | 0.91 (0.79; 1.03) | 0.142 |  |  |
| *Never smoked* | 0.92 (0.74; 1.14) | 0.445 |  |  |
| Bladder | 0.89 (0.75; 1.05) | 0.175 | 0.92 (0.78; 1.09) | 0.325 |
| Kidney | **0.81 (0.71; 0.92)** | **0.001** | **0.81 (0.71; 0.93)** | **0.002** |
| Pancreas | **0.87 (0.77; 0.99)** | **0.038** | 0.88 (0.77; 1.01) | 0.060 |
| Uterus | 0.92 (0.81; 1.05) | 0.234 | 0.92 (0.80; 1.05) | 0.196 |
| Oesophagus | **0.81 (0.69; 0.95)** | **0.009** | **0.83 (0.71; 0.98)** | **0.025** |
| *Current smoker* | **0.64 (0.42; 0.99)** | **0.043** |  |  |
| *Previous smoker* | 0.81 (0.64; 1.02) | 0.069 |  |  |
| *Never smoked* | 0.97 (0.74; 1.26) | 0.814 |  |  |
| Head and Neck | 1.17 (0.99; 1.39) | 0.032 | **1.21 (1.01; 1.44)** | **0.034** |
| *Current smoker* | 1.13 (0.70; 1.84) | 0.615 |  |  |
| *Previous smoker* | 1.23 (0.96; 1.58) | 0.102 |  |  |
| *Never smoked* | 1.19 (0.90; 1.58) | 0.227 |  |  |
| Ovary | **0.77 (0.66; 0.90)** | **0.001** | **0.77 (0.66; 0.90)** | **0.001** |
| Stomach | 0.94 (0.77; 1.14) | 0.518 | 0.95 (0.78; 1.16) | 0.604 |
| Liver | **0.77 (0.63; 0.96)** | **0.019** | **0.78 (0.63; 0.97)** | **0.025** |
| Gallbladder | **0.71 (0.54; 0.94)** | **0.018** |  |  |

Data are presented as hazard ratio (HR) with 95% confidence intervals in parentheses (95% CIs) per 1-point increment in score. Model 1 was adjusted for age, sex, Townsend deprivation index and ethnicity. Model 2 was additionally adjusted for smoking status.

**Table S10: Sensitivity analysis for associations between total score using original cut-points (based on US guidelines) for alcohol score component, categorised according to score tertiles, and risk of all cancers and of cancers at individual anatomical sites**

|  |  |  | **Low score (0-3.5)** | **Higher score (4.5 – 7)** | | | |
| --- | --- | --- | --- | --- | --- | --- | --- |
| **Cancer site** | **Total** | **Events** | **HR (95% CI)** | **Model 1 HR (95% CI)** | **P value** | **Model 2 HR (95% CI)** | **P value** |
| All cancers combined | 93,630 | 7,296 | 1.00 (ref) | **0.84 (0.79; 0.96)** | **<0.001** | **0.85 (0.80; 0.90)** | **<0.001** |
| Prostate | 43,851 | 1,818 | 1.00 (ref) | 1.01 (0.90; 1.13) | 0.973 |  |  |
| Breast | 50,337 | 1,438 | 1.00 (ref) | **0.83 (0.73; 0.93)** | **0.002** | **0.83 (0.73; 0.94)** | **0.002** |
| *Pre-Menopausal* | 1,108 | 154 | 1.00 (ref) | 0.74 (0.52; 1.06) | 0.105 | 0.74 (0.52; 1.06) | 0.100 |
| *Post-Menopausal* | 49,229 | 1,284 | 1.00 (ref) | **0.82 (0.72; 0.93)** | **0.002** | **0.82 (0.72; 0.93)** | **0.002** |
| Colorectal | 94,656 | 862 | 1.00 (ref) | **0.77 (0.65; 0.90)** | **0.001** | **0.79 (0.67; 0.92)** | **0.004** |
| *Colon* | 94,698 | 575 | 1.00 (ref) | **0.65 (0.53; 0.79)** | **<0.001** | **0.66 (0.54; 0.81)** | **<0.001** |
| *Distal* | 94,734 | 242 | 1.00 (ref) | **0.53 (0.38; 0.73)** | **<0.001** | **0.54 (0.39; 0.74)** | **<0.001** |
| *Proximal* | 94,748 | 303 | 1.00 (ref) | **0.74 (0.56; 0.97)** | **0.028** | **0.75 (0.57; 0.99)** | **0.040** |
| *Rectum* | 94,734 | 342 | 1.00 (ref) | 1.01 (0.79; 1.30) | 0.922 | 1.06 (0.82; 1.36) | 0.676 |
| Lung | 94,760 | 431 | 1.00 (ref) | **0.68 (0.54; 0.86)** | **0.001** | 0.81 (0.64; 1.02) | 0.074 |
| *Current smoker* | 6,387 | 125 | 1.00 (ref) | 0.90 (0.57; 1.42) | 0.658 |  |  |
| *Previous smoker* | 33,260 | 225 | 1.00 (ref) | 0.72 (0.52; 1.00) | 0.053 |  |  |
| *Never smoked* | 54,971 | 80 | 1.00 (ref) | 0.89 (0.53; 1.48) | 0.648 |  |  |
| Kidney | 94,754 | 221 | 1.00 (ref) | **0.62 (0.44; 0.87)** | **0.005** | **0.62 (0.44; 0.88)** | **0.007** |
| Pancreas | 94,770 | 213 | 1.00 (ref) | 0.74 (0.54; 1.02) | 0.067 | 0.76 (0.55; 1.05) | 0.098 |
| Uterus | 50,588 | 212 | 1.00 (ref) | 0.84 (0.61; 1.17) | 0.305 | 0.83 (0.60; 1.15) | 0.266 |
| Oesophagus | 94,765 | 151 | 1.00 (ref) | **0.59 (0.39; 0.90)** | **0.014** | **0.64 (0.42; 0.96)** | **0.033** |
| *Current smoker* | 6,388 | 22 | 1.00 (ref) | 0.38 (0.09; 1.65) | 0.473 |  |  |
| *Previous smoker* | 33,260 | 73 | 1.00 (ref) | 0.55 (0.30; 1.01) | 0.055 |  |  |
| *Never smoked* | 54,975 | 56 | 1.00 (ref) | 0.89 (0.47; 1.70) | 0.728 |  |  |
| Ovary | 50,603 | 147 | 1.00 (ref) | **0.59 (0.40; 0.87)** | **0.007** | **0.59 (0.40; 0.87)** | **0.007** |
| Bladder | 94,756 | 141 | 1.00 (ref) | **0.65 (0.43; 0.99)** | **0.045** | 0.70 (0.46; 1.06) | 0.094 |
| Head and Neck | 94,763 | 129 | 1.00 (ref) | 1.43 (0.94; 2.17) | 0.097 | 1.52 (1.00; 2.32) | 0.052 |
| *Current smoker* | 6,388 | 16 | 1.00 (ref) | 1.54 (0.48; 4.93) | 0.467 |  |  |
| *Previous smoker* | 33,259 | 63 | 1.00 (ref) | 1.39 (0.76; 2.55) | 0.287 |  |  |
| *Never smoked* | 54,974 | 50 | 1.00 (ref) | 1.66 (0.84; 3.28) | 0.148 |  |  |
| Stomach | 94, 770 | 99 | 1.00 (ref) | 0.88 (0.55; 1.43) | 0.607 | 0.91 (0.56; 1.47) | 0.689 |
| Liver | 94,773 | 82 | 1.00 (ref) | 0.71 (0.42; 1.19) | 0.189 | 0.73 (0.43; 1.22) | 0.227 |
| Gallbladder | 94,777 | 49 | 1.00 (ref) | 0.57 (0.28; 1.14) | 0.113 |  |  |

Data are presented as hazard ratio (HR) with 95% confidence intervals in parentheses (95% CIs). Lowest score tertile was the reference group. Model 1 was adjusted for age, sex, Townsend deprivation index and ethnicity. Model 2 was additionally adjusted for smoking status.

**Table S11: Sensitivity analysis for associations between total score and risk of all cancers and of cancers at individual anatomical sites using the date of completion of last 24-hour dietary assessment as baseline**

| **Cancer site** | **Total** | **Events** | **Continuous score**  **HR (95% CI)** | **p-value** | **Low score**  **HR (95% CI)** | **Middle score**  **HR (95% CI)** | **P value** | **Higher score**  **HR (95% CI)** | **P value** |
| --- | --- | --- | --- | --- | --- | --- | --- | --- | --- |
| All cancers combined | 92,941 | 6,758 | 0.93 (0.90; 0.95) | <0.001 | 1.00 (ref) | **0.91 (0.86; 0.97)** | **0.002** | **0.83 (0.78; 0.88)** | **<0.001** |
| Prostate | 43,627 | 1,668 | 0.99 (0.95; 1.04) | 0.822 | 1.00 (ref) | 1.01 (0.91; 1.14) | 0.809 | 0.95 (0.84; 1.08) | 0.424 |
| Breast | 50,107 | 1,290 | **0.91 (0.86; 0.96)** | **0.001** | 1.00 (ref) | **0.84 (0.73; 0.97)** | **0.016** | **0.84 (0.74; 0.95)** | **0.007** |
| *Pre-Menopausal* | 1,072 | 121 | 0.85 (0.72; 1.00) | 0.055 | 1.00 (ref) | **0.60 (0.37; 0.96)** | **0.034** | **0.66 (0.44; 0.99)** | **0.048** |
| *Post-Menopausal* | 49,035 | 1,169 | **0.91 (0.86; 0.97)** | **0.002** | 1.00 (ref) | **0.86 (0.74; 0.99)** | **0.039** | **0.84 (0.74; 0.97)** | **0.014** |
| Colorectal | 94,417 | 788 | **0.91 (0.84; 0.97)** | **0.005** | 1.00 (ref) | **0.78 (0.66; 0.93)** | **0.005** | **0.77 (0.65; 0.92)** | **0.004** |
| *Colon* | 94,486 | 527 | **0.83 (0.77; 0.91)** | **<0.001** | 1.00 (ref) | **0.71 (0.58; 0.88)** | **0.002** | **0.67 (0.54; 0.83)** | **<0.001** |
| *Distal* | 94,544 | 216 | **0.75 (0.66; 0.85)** | **<0.001** | 1.00 (ref) | **0.65 (0.46; 0.90)** | **0.010** | **0.54 (0.38; 0.76)** | **0.001** |
| *Proximal* | 94,563 | 283 | 0.90 (0.80; 1.01) | 0.078 | 1.00 (ref) | 0.81 (0.61; 1.07) | 0.142 | 0.76 (0.57; 1.01) | 0.060 |
| *Rectum* | 94,542 | 315 | 1.03 (0.92; 1.15) | 0.658 | 1.00 (ref) | 0.91 (0.69; 1.19) | 0.488 | 1.01 (0.77; 1.32) | 0.967 |
| Lung | 94,585 | 420 | 0.92 (0.83; 1.01) | 0.083 | 1.00 (ref) | 0.98 (0.78; 1.23) | 0.868 | **0.78 (0.61; 0.99)** | **0.043** |
| Bladder | 94,584 | 134 | 0.92 (0.78; 1.09) | 0.349 | 1.00 (ref) | 0.76 (0.50; 1.15) | 0.198 | 0.74 (0.47; 1.15) | 0.181 |
| Kidney | 94,573 | 205 | **0.82 (0.71; 0.94)** | **0.004** | 1.00 (ref) | 0.88 (0.63; 1.21) | 0.420 | **0.64 (0.45; 0.93)** | **0.018** |
| Pancreas | 94,599 | 207 | **0.81 (0.70; 0.92)** | **0.002** | 1.00 (ref) | **0.71 (0.51; 0.99)** | **0.046** | **0.60 (0.42; 0.84)** | **0.003** |
| Uterus | 50,481 | 190 | 0.90 (0.78; 1.03) | 0.138 | 1.00 (ref) | 0.99 (0.69; 1.40) | 0.938 | 0.75 (0.53; 1.06) | 0.100 |
| Oesophagus | 94,598 | 149 | **0.82 (0.70; 0.96)** | **0.016** | 1.00 (ref) | 0.82 (0.56; 1.21) | 0.318 | **0.60 (0.39; 0.93)** | **0.023** |
| Head and Neck | 94,595 | 126 | **1.23 (1.04; 1.47)** | **0.019** | 1.00 (ref) | 1.54 (0.99; 2.38) | 0.050 | **1.59 (1.03; 2.45)** | **0.036** |
| Ovary | 50,508 | 136 | **0.78 (0.66; 0.92)** | **0.004** | 1.00 (ref) | 0.65 (0.43; 1.00) | 0.052 | **0.64 (0.43; 0.94)** | **0.023** |
| Stomach | 94,603 | 96 | 0.91 (0.75; 1.12) | 0.374 | 1.00 (ref) | 0.93 (0.57; 1.51) | 0.770 | 0.87 (0.52; 1.44) | 0.576 |
| Liver | 94,606 | 80 | **0.79 (0.63; 0.98)** | **0.032** | 1.00 (ref) | 0.79 (0.46; 1.36) | 0.399 | 0.74 (0.43; 1.29) | 0.286 |
| Gallbladder | 94,611 | 48 | **0.69 (0.52; 0.92)** | **0.011** | 1.00 (ref) | 0.61 (0.30; 1.24) | 0.174 | 0.54 (0.26; 1.11) | 0.093 |

Data are presented as hazard ratio (HR) with 95% confidence intervals in parentheses (95% CIs). Lowest score tertile was the reference group. Results represent those for model 2 adjusted for age, sex, Townsend deprivation index ethnicity, and smoking status.
